# Studies of Coumarin Derivatives for Constitutive Androstane Receptor (CAR) Activation

**DOI:** 10.3390/molecules26010164

**Published:** 2020-12-31

**Authors:** Shin-Hun Juang, Min-Tsang Hsieh, Pei-Ling Hsu, Ju-Ling Chen, Hui-Kang Liu, Fong-Pin Liang, Sheng-Chu Kuo, Chen-Yuan Chiu, Shing-Hwa Liu, Chen-Hsi Chou, Tian-Shung Wu, Hsin-Yi Hung

**Affiliations:** 1 School of Pharmacy, China Medical University, Taichung 404, Taiwan; Paul.juang@gmail.com (S.-H.J.); t21917@mail.cmu.edu.tw (M.-T.H.); Peiling96@livemail.tw (P.-L.H.); ericababa@msn.com (J.-L.C.); cellpharmacology@gmail.com (F.-P.L.); sckuo@mail.cmu.edu.tw (S.-C.K.); 2 Chinese Medicine Research and Development Center, China Medical University Hospital, 2 Yude Road, Taichung 404, Taiwan; 3 School of Pharmacy, College of Medicine, National Cheng Kung University, Tainan 701, Taiwan; chenhsi@mail.ncku.edu.tw (C.-H.C.); tswu@mail.ncku.edu.tw (T.-S.W.); 4 National Research Institute of Chinese Medicine, Ministry of Health and Welfare, Taipei 112, Taiwan; hk.liu@nricm.edu.tw; 5 Graduate Institute of Toxicology, College of Medicine, National Taiwan University, Taipei 100, Taiwan; kidchiou@gmail.com (C.-Y.C.); shinghwaliu@ntu.edu.tw (S.-H.L.)

**Keywords:** Yin Chen Hao, constitutive androstane receptor, coumarin, scoparone

## Abstract

Constitutive androstane receptor (CAR) activation has found to ameliorate diabetes in animal models. However, no CAR agonists are available clinically. Therefore, a safe and effective CAR activator would be an alternative option. In this study, sixty courmarin derivatives either synthesized or purified from *Artemisia capillaris* were screened for CAR activation activity. Chemical modifications were on position 5,6,7,8 with mono-, di-, tri-, or tetra-substitutions. Among all the compounds subjected for in vitro CAR activation screening, 6,7-diprenoxycoumarin was the most effective and was selected for further preclinical studies. Chemical modification on the 6 position and unsaturated chains were generally beneficial. Electron-withdrawn groups as well as long unsaturated chains were hazardous to the activity. Mechanism of action studies showed that CAR activation of 6,7-diprenoxycoumarin might be through the inhibition of EGFR signaling and upregulating PP2Ac methylation. To sum up, modification mimicking natural occurring coumarins shed light on CAR studies and the established screening system provides a rapid method for the discovery and development of CAR activators. In addition, one CAR activator, scoparone, did showed anti-diabetes effect in *db*/*db* mice without elevation of insulin levels.

## 1. Introduction

Constitutive androstane receptor (CAR, NR1I3), a member of the superfamily of nuclear receptors, is a xenobiotic receptor responsible for the regulation of drug metabolism as well as the pathological involvement of various diseases such as cancer, diabetes, inflammatory disease, metabolic disease and liver disease, suggesting a potential target for drug discovery [1]. Predominantly expressed in the liver, once CAR becomes activated, it disassociates from the cytoplasmic protein complex and with retinoid X receptor (RXR) binds to specific DNA region and influence target gene expression [2].

CAR has been proven to involve in various energy pathways as well as metabolic pathways [3]. The well-established function of CAR is to regulate bile acid detoxification and bilirubin clearance via the induction of metabolizing enzymes and transporters, such as Mrp2–4, Oatp 2, Cyp3a11, Sult2a1 and Ugt1a1 [4,5,6]. Moreover, CAR knock out (KO) mice led to high incidence of hepatic necrosis and high level of alanine aminotransferase (ALT) and bilirubin, suggesting an important function of CAR activation for hepatoprotective effect and a potential role in treating cholestasis [7]. A direct CAR agonist, TCPOBOP, was found to decrease liver injury and hepatocyte apoptosis in the treated mice as well as to improve insulin sensitivity in ob/ob mice model [8]. In addition, CAR activation ameliorates hyperglycemia as well as fatty liver by suppressing glucose production, stimulating glucose uptake and usage in the liver and inhibiting hepatic lipogenesis and induction of β–oxidation [9]. Recent metabolomic research revealed CAR activation significantly lower mRNA expression that involved in gluconeogenic pathway, up-regulate glucose utilization pathways, enhance specific fatty acid synthesis and impair β–oxidation [10].

**Figure 1:**
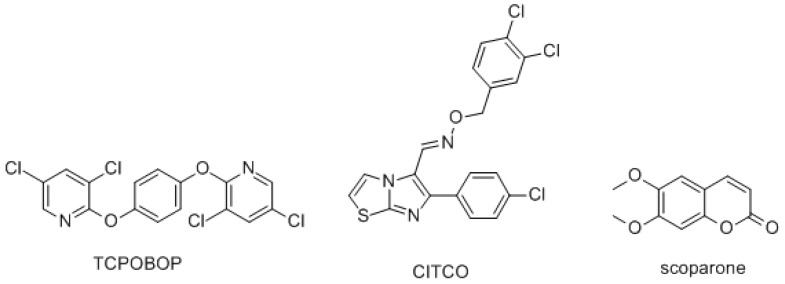
Structures of CAR direct activators. TCPOBOP is a mouse CAR direct activator. CITCO is a human CAR direct activator.

Since CAR is a potential target for various diseases, neither CAR agonists nor antagonists are seen clinically. 6-(4-Chlorophenyl) imidazo [2,1-b] [1,3] thiazole-5-carbaldehyde *O*-(3,4-dichlorobenzyl) oxime (CITCO), a human CAR agonist, is used extensively in biological studies, but failed to be further developed due to toxicity. Another commonly used agonist, TCPOBOP (Figure 1), is a mouse CAR agonist, which also points out the difficulty of developing CAR activators—species specificity [11]. Luckily, Yin Chen Hao (*Artemisia capillaris*) in the Asteraceae family has long been used to treat jaundice in Traditional Chinese Medicine (TCM). The extract of Yin Chen Hao Tang (YCHT) was found to activate CAR and then accelerated bilirubin clearance in animal models [12,13]. In 2015, a meta-analysis studied 15 randomized controlled trials (RCT) including 1405 cases ranging from 2000 to 2014 and found YCHT reduced the elevated levels of cholestasis serum markers significantly either in short or long curative time periods and also improved the clinical efficiency of other anti-cholestasis medications [14]. Further, 6,7-dimethylesculetin (scoparone), a major active principal isolated from Yin Chin Hao, also exerted CAR activation activity both in vitro and in vivo [12]. Scoparone exerted plenty of pharmacological activites, such as hepatoprotective effect, antioxidation, anti-inflammation, and anti-cholestasis [15]. In addition, scoparone (10 μM) potentiated chenodeoxycholic acid (10 μM, a potent FXR agonist) effect to increase the expression of bile salt export pump. However, scoparone alone (up to 100 μM) had no effect on FXR activation [16].

Inspired by the findings above, this study was designed to mimic coumarins from Yin Chen Hao, to systemically synthesize coumarin derivatives to study structure-activity relationship of coumarin derivatives on CAR activation and to establish in vitro CAR activation screening method for discovering and developing therapeutic CAR activation agents in the future. Further, the mechanism of action of the most active compound will be discussed and a possible indication, anti-diabetes, of a CAR activator will be evaluated in vivo.

## 2. Results and Discussion

### Chemistry

Although coumarin derivatives have been studied extensively, their effects on CAR activation are largely underinvestigated. In coumarins isolated from Yin Chen Hao, all the substitutions are on C5, C6, C7, C8 positions [17]. Since the exact binding site of scoparone is unclear, ligand-based drug design is applied. The first strategy is to evaluate the substitution effect on C5, C6, C7, C8 positions alone and together to establish SAR (Table 1, Table 2 and Table 3). Various alkoxy groups including different chain length, branch, and unsaturation were synthesized and studied in these four positions. These modifications included the structures of natural coumarins, such as 5-hydroxycoumarin, 5-methoxycoumarin, umbeliferone, methylumbeliferone, aurapten, 8-hydroxycoumarin, 8-methoxycoumarin, esculetin, isoscopoletin, scopoletin, scoparone, ayapin, isoscopolin, scopolin, fraxinol, and leptodactylone. Their natural occurrence is provided in the reference column of the tables. From the references, we can find these coumarins can be found in many clinically used TCMs or herbs. Moreover, we also synthesized eight new derivatives (compounds **11**, **20**, **44**, **46–49**, **50**) for comparison of SAR. The syntheses of the coumarin derivatives were mainly via Perkin reaction [18] or Knoevenagel reaction [19] or Pechmann reaction [20] or Wittig reactions [21,22] or others [23,24] and the spectral data for the known compounds were identical with the literature values.

Moreover, syntheses of coumarins disubstituted mainly on the C6 and C7 positions were carried out (Table 2) The starting material, 6,7-dihydroxycoumarin, was commercially available. Various alkoxyl substitutions were synthesized by treating 6,7-dihydroxycoumarin in dimethylformamide with the corresponding alkyl bromide. Tri-and tetrasubstituted coumarins were also synthesized and are summarized in Table 3. Scopolin (**53**), isoscopolin (**52**), compound **59** and **60** were isolated from Yin Chen Hao [17].

## 3. Establishment of In Vitro CAR Activation Screening Assay

**Figure 2:**
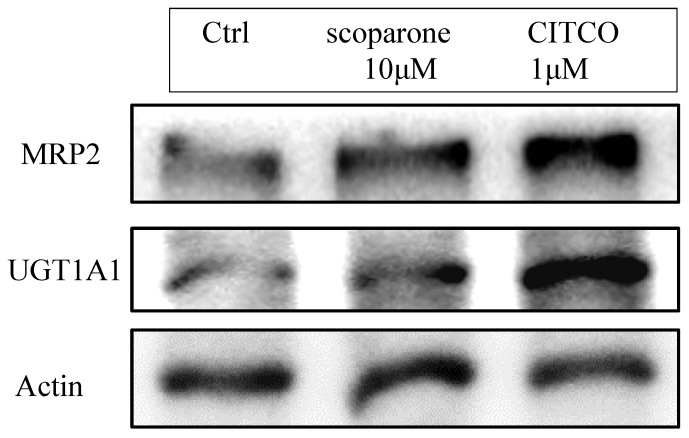
CAR downstream signal protein expression after treatment of CITCO or scoparone.

Until now, there is no commercially available high throughput screening method for CAR activation. Traditionally, CAR activation can be detected in western blots by its downstream signals such as UGT1A1, MRP2 or CYP2B6. In Figure 2, we confirmed that the protein expressions of downstream genes of CAR, UGT1A and MRP2, were upregulated after scoparone and CITCO treatment. However, this method was slow and pricy. In order to rapidly screen all the compounds for CAR activation at less cost, an in vitro CAR activation screening assay was developed: DNA was extracted from Hep3B cells and UGT1A1 promoter was obtained using PCR amplification. Ligation between UGT1A1 promoter and pGL4 vector was performed and the plasmid was amplified via *E. coli* replication. The desired plasmid was extracted from E. coli and transfected into Hep3B. The desired colony bearing UGT1A1 promoter-pGL4 plasmid was selected by neomycin. CAR activation was measured by adding tested compounds to Hep3B for several hours and luminescence was measured. Higher activation will get higher luminescence readout. This assay system was validated with two different CAR agonists: CITCO (direct agonist) and scoparone (an indirect agonist). CITCO could induce about 50% elevation of luciferase activity while scoparone as a positive control could enhance luciferase activity too. Different concentration of scoparone at 1, 5, 10 and 20 μM had been tested either for luciferase assay or downstream UGT1A1 and MRP2 expression (data not shown) and 10 μM was selected to be the best condition for the following work.

After successfully establishing the in vitro assay, all of the coumarin compounds were subjected to the screening (at 10 μM) and their SAR was discussed. Although the reporter system can successfully identify CAR-activating compounds under 8 h and display better efficiency and sensitivity compared with western blots, it only presented a medium luciferase response. According to previous studies, CITCO was reported to only moderately enhance human CAR (less than 2-fold), compared with the enhance provided by TCPOBOP for murine CAR (5- to 10-fold) in luciferase reporter assays [11,54]. These results suggested that human CAR might have a weaker response to agonists compared to murine CAR, which might be the same situation as in our reporter system. Another possibility for the medium response in our reporter system might be the contribution of the whole gtPBREM element which might include response-suppressive elements that was used and the single nucleotide variation of DR3 element which was reported to slightly reduce the original UGT1A1 activity [55].

## 4. Structure-Activity Relationships

All the compounds were compared to scoparone (**39**) and the resulting CAR activation fold results (%) are shown in Table 1, Table 2 and Table 3. This is the readout of the tested compound minus the readout of scoparone and then divided by the scoparone readout. Among all sixty compounds, twenty compounds exhibited better or similar CAR activation ability than scoparone and 6,7-diprenoxycoumarin (**50**) was the best compound with a 50% increased CAR activation fold. In addition, none of the compounds have any cytotoxicity at this concentration (IC_50_ > 50 μM) except for **47** (IC_50_ = 31 μM). As for a detailed SAR discussion of the mono- and disubstituted coumarins, hydroxyl groups except on position 8 exhibited similar or less activity than scoparone. Methoxy substitution is better on position 6 than positions 5, 7 or 8. Electron withdrawing groups, such as an acetyl group or a trifluoromethyl group decrease the activity, suggesting the importance of enough electrons. A medium carbon chain length such as a *n*-butyl group on the 6 position is good. Prenyl groups are general good, except in the 7 position. Longer unsaturated chains such as geranyl or farnesyl reduce the activity. To sum up, electron withdrawing groups would be detrimental to the activity. Modification on the 6 position generally increases the activity. Longer unsaturated chains are not good for the activity. Among tri- and tetra-substituted coumarins, 5,7-dimethoxy-8-hydroxycoumarin (**57**) and 6,7,8-trimethoxycoumarin (**58**) showed comparable CAR activation effects to scoparone. 6-Methoxy-7,8-methylenedioxycoumarin (**59**) exhibited a good activation effect (36% increase) and 5,6-dimethoxy-7,8- methylenedioxycoumarin (**60**) also had good activity.

Moreover, if we examined coumarins reported to exist in Yin Chen Hao, such as esculetin (**35**), isoscopoletin (**37**), scopoletin (**38**), scoparone (**39**), scopolin (**53**), isoscopolin (**52**), 5,6,7-trimethoxycoumarin (**54**), leptodactylone (**57**), compound **59** and **60**, we can find that compounds **52**, **53**, **57**, **59** and **60** had superior CAR activating activity than scoparone, implying that the anti-cholestasis effect of YCHT might be an added effect of all the coumarins inside.

## 5. Mechanistic Studies of 6,7-Diprenoxycoumarin (50)

**Figure 3:**
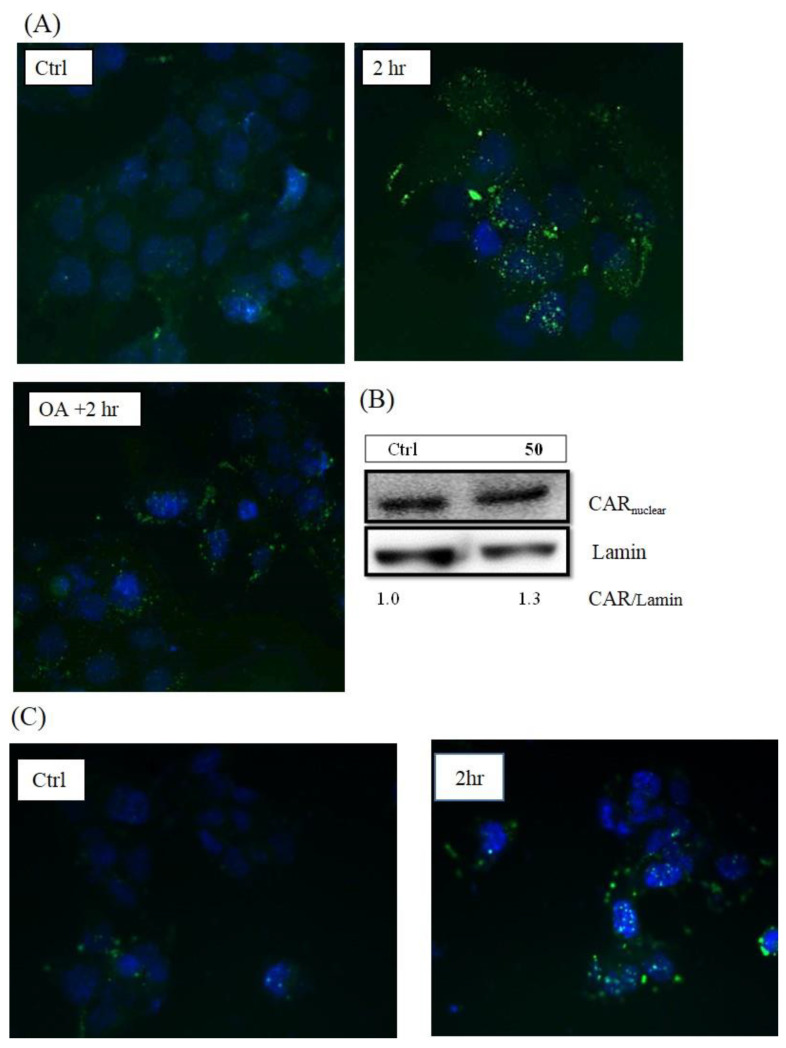
Image of immunofluorescence staining for CAR translocation in Hep3B cells after 6,7-diprenoxycoumarin (**50**) treatment (**A**) and scoparone treatment (**C**). Hep3B cells were treated with **50** (10 μM) for 2 h. The negative control group was pre-treated with 10 nM okadaic acid (OA). The blue and green fluorescence stands for signals of nuclei and CAR protein, respectively. The protein expression of nuclear CAR after 6,7-diprenoxycoumarin (**50**) treatment for 2 h (**B**).

First, the translocation of CAR protein was confirmed after treatment with **50** (Figure 3A), and the translocation activity could be inhibited by okadaic acid (OA), the phosphatase inhibitor which inhibits CAR from translocating into the nucleus. In addition, the protein expression of nuclear CAR increased after **50** treatment as shown in Figure 3B.

**Figure 4:**
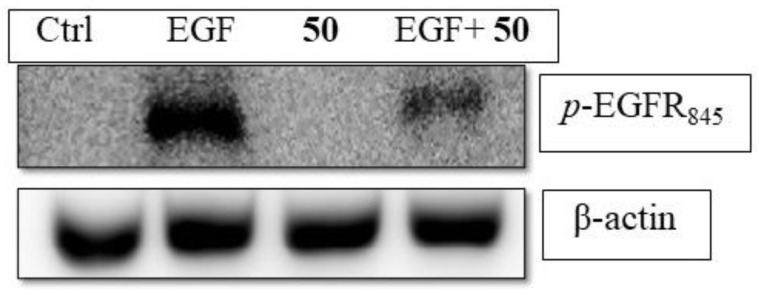
6,7-Diprenoxycoumarin (**50**) antagonized EGF-induced phosphorylation EGFR in Hep3B. The Hep3B cells (1 × 10^6^) were treated without (Ctrl) or with EGF in the absent **50** or present (EGF + **50**) of 10 μM **50** for 15 min and phosphorylation status of EGFR was measured.

**Figure 5:**
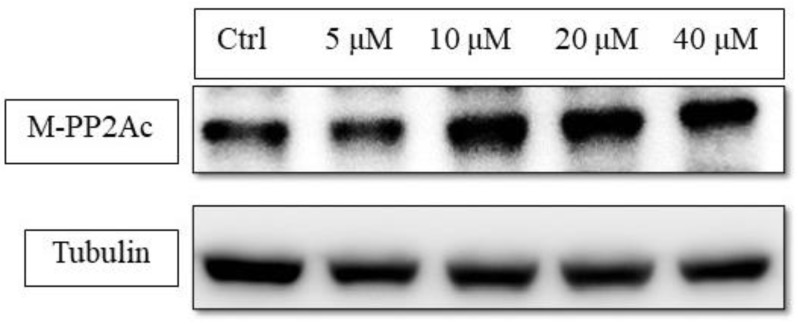
The concentration-dependent study of PP2Ac methylation status under 6,7-diprenoxycoumarin (**50**) treatment. Hep3B cells (1 × 10^6^) were treated with **50** (5, 10, 20 and 40 μM) for 24 h. Treated cell lysates were prepared as described in the Methods section and methyl-PP2Ac expression was analyzed by western blot.

Like phenobarbital-mediated CAR activation, **50** activated CAR through inhibiting EGFR phosphorylation [56]. Western blot results indicated that **50** could reduce EGF-induced phosphorylation of EGFR in Hep3B (Figure 4). The up-stream protein, PP2Ac, which is responsible for CAR dephosphorylation was increased with its active form, methyl-PP2Ac (Figure 5).

**Figure 6:**
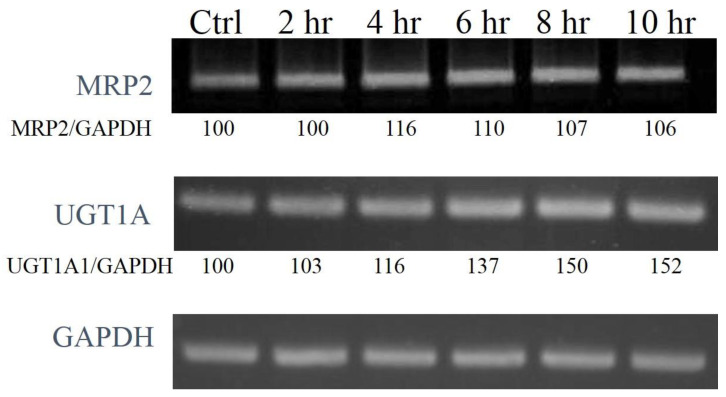
The time course study of mRNA expression of MRP2 and UGT1A1 after 10 μM 6,7-diprenoxycoumarin treatment. Hep3B cells were treated with 10 μM **50** for 2, 4, 6, 8 and 10 h. The total mRNA was obtained and the expression of downstream genes were analyzed with PCR. The ratio of MRP2 to GAPDH was calculated using Image J and normalization.

**Figure 7:**
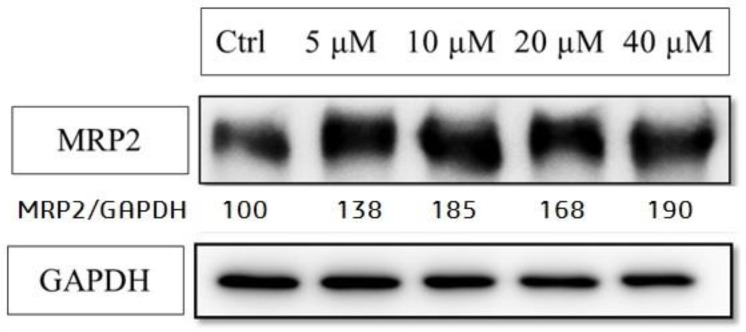
The protein expressions of MRP2 after 6,7-diprenoxycoumarin (**50**) treatment. Hep3B cell (1 × 10^6^) were treated with **50** (5, 10, 20 and 40 μM) for 24 h. Treated cell lysates were prepared as described in the Methods section and the expression of MRP2 was analyzed by western blot.

The mRNA and protein expression level of CAR-related down-stream genes, MRP2 and UGT1A1, were also enhanced by **50** (UGT1A1 protein level was not significantly increased in our study) (Figure 6 and Figure 7). These data strongly supported that the CAR activation of coumarin derivatives might through the inhibiting the EGFR signaling and upregulating PP2Ac methylation.

## 6. Further In Vivo Hypoglycemic Effect and Pharmacokinetic Studies

**Figure 8:**
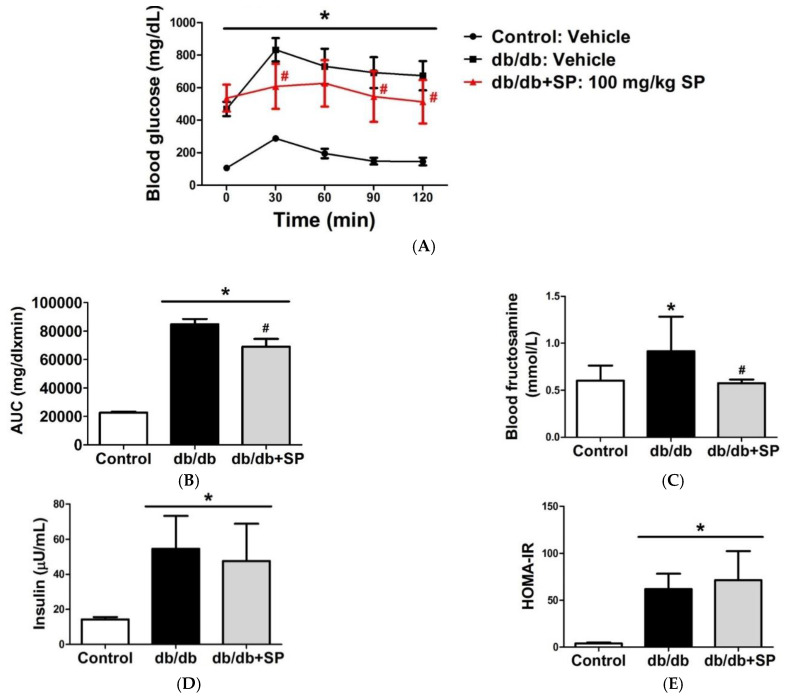
Treatment of scoparone on db/db mice for two weeks improves glucose tolerance and insulin sensitivity. Treatment of scoparone on db/db mice for two weeks improves glucose tolerance. (**A**) db/db mice were treated with scoparone or vehicle (P.O.) for 2 weeks. After 12 h of fasting, glucose tolerance tests were performed and blood glucose levels were assessed (*n* = 6) (**B**) Area under curve of blood glucose level were calculated. Fructosamine (**C**) and insulin (**D**) levels were measured. (**E**) Homeostasis Model Assessment (HOMA) was calculated as HOMA-IR index = insulin(μU/mL) × glucose(mmol/L)/22.5. (* statistically significant compared with Control group; # statistically significant compared with db/db group; Probability of < 0.05 was considered to be significant)

From previous literature, CAR activator has been found to regulate glucose metabolizing gene expression [9]. Therefore, in vivo hypoglycemic effect was evaluated using scoparone and compound **50** (Figure 8). Db/db mice were treated with scoparone (100 mg/kg, P.O.) or vehicle (CMC) for two weeks. The scoparone-treated group showed a significant improvement in oral glucose tolerance test (OGTT), which was not observed in vehicle group (A). Further analysis showed the level of fructosamine, a glycation end product, also decreased after treatment, indicating blood glucose levels became lower after treatment (B). However, the insulin level (C) or HOMA-IR (D) did not change, indicating that glucose-lowering effect was not due to an insulinotropic effect. Compound **50** didn’t show any in vivo efficacy even by intraperitoneal injection, which led us to perform a pharmacokinetic study of scoparone and **50** (Supplementary Figure S21). After analytical method validation, C57BL/6 mice were given scoparone (i.p.) or **50** and the resulting plasma concentration-time are presented in Tables S1 and S2. In the pharmacological assay, the desired scoparone concentration is ~2 μg/mL (10 μM), which can be achieved when the dose given is 0.5 mg/mouse (Table S1). However, the desired concentration of **50** (~3 μg/mL, 10 μM) cannot be obtained even if the amount of drug is increased to 0.5 mg/mice. Thus, the CAR activating effect of **50** may not be seen in vivo, which can account for the fact no blood sugar lowering effects of **50** were observed in vivo.

## 7. Conclusions

CAR activation has already been associated with amelioration of various diseases such as diabetes. However, clinical CAR agonists are still lacking, hterefore, a safe and effective CAR activator would be an interesting alternative option for these diseases. In this study, sixty coumarin derivatives either synthesized or purified from Yin Chen were screened for CAR activation activity. Among all the compounds, **50** is the most effective for CAR activation and was selected for further studies. Modification on the 6 position is generally more beneficial than at other positions. Electron-withdrawn groups are detrimental to the activity. Mechanism of action studies showed that CAR activation of **50** might be through the inhibition of EGFR signaling and upregulated PP2Ac methylation. In vivo OGTT, scoparone can lower blood sugar and fructosamine levels without insulinotropic effects. In addition, a preliminary pharmacokinetic study also indicated the desired scoparone blood concentration can be achieved. This study established also a screening system providing a rapid method for CAR activator discovery and development and provided scientific evidence for the effects of coumarins on CAR activation.

## 8. Materials and Methods

### 8.1. General Information

All of the solvents and reagents were obtained commercially and used without further purification. The progress of all the reactions was monitored by TLC on 2 × 6 cm pre-coated silica gel 60 F_254_ plates of thickness 0.25 mm (Merck). The chromatograms were visualized under 254 or 366 nm UV light. Silica gel 60 (Merck, particle size 0.063–0.200 mm) was used as the adsorbant for column chromatography.

NMR spectra were obtained on an AV400 NMR spectrometer (Bruker). MS spectra were measured either at the instruments center of National Chung Hsing University (JMS-700 spectrometer, JEOL, Japan) or National Tsing Hua University (MAT-95XL HRMS, Finnigan, San Jose, CA). CITCO was purchased from Sigma-Aldrich (Oakville, ON, Canada).

### 8.2. Chemistry

*6-Trifluoromethoxycoumarin* (**11**). 2-Hydroxy-4-trifluoromethoxylbenzaldehyde (0.5 g, 2.4 mmol) and methoxycarbonylmethylene-triphenylphosphorane (0.9 g, 2.8 mmol) were dissolved in *N*, *N*-diethylaniline (5.0 mL) and refluxed for 4 h. The reaction mixture was purified by silica gel column chromatography (*n*-hexane/ethyl acetate (8:2)) to give the intermediate. Then the intermediate was dissolved in diphenyl ether (5.0 mL), heated to 200 °C for 4 h. The mixture was partitioned with water and dichloromethane and dried over MgSO_4_. The desired compound was obtained by silica gel column chromatography (*n*-hexane/ethyl acetate (8:2)) to give 6-trifluoromethoxycoumarin (**11**, 0.3 g, 1.3 mmol). Yellow needle-like crystals. Yield: 55.0%. M.P.: 83–85 °C. ^1^H-NMR (400 MHz, CDCl_3_): δ 7.71 (1H, d, *J* = 9.6 Hz), 7.43–7.38 (2H, m), 7.28 (1H, s), 6.53 (1H, d, *J* = 9.6 Hz). ^13^C-NMR (100 MHz, CDCl_3_): δ 159.8, 152.1, 145.0, 142.3, 124.7, 120.3 (q, *J* = 258 Hz), 119.7, 119.4, 118.3, 117.9. ^19^F-NMR (470 MHz, CDCl_3_): δ = −58.31. HRMS [ESI]^+^ calculated for C_10_H_5_F_3_O_3_: 230.0191; found [M]^+^ 230.0192.

*7-Trifluoromethoxycoumarin* (**20**). 3-Trifluoromethoxylphenol (1.0 g, 5.6 mmol), palladium (II) acetate (5.0 mol%) and trifluoroacetic acid (8.4 mL) were mixed in dichloromethane (5.0 mL) under nitrogen and stirred at room temperature for 7 days. Brine solution was added and the misture extracted with ethyl acetate. The ethyl acetate layer was washed with brine and dried over anhydrous MgSO_4_. The solvent was removed in vacuo. The residue was purified by column chromatography (*n*-hexane/ethyl acetate (7:3)) to give 7-trifluoromethoxycoumarin (**20**, 0.06 mg, 0.3 mmol) and recovered starting material. White crystals. Yield: 4.0%. M.P.: 69–71 °C. ^1^H-NMR (400 MHz, CDCl_3_): δ 7.68 (1H, d, *J* = 9.6 Hz), 7.50 (1H, d, *J* = 8.4 Hz), 7.15 (1H, s), 7.11 (1H, d, *J* = 8.4 Hz), 6.24 (1H, d, *J* = 9.6 Hz). ^13^C- NMR (100 MHz, CDCl_3_): δ 159.7, 154.6, 151.2, 142.4, 129.0, 120.1 (q, *J* = 258 Hz), 117.2, 116.7, 116.6, 109.1. ^19^F-NMR (470 MHz, CDCl_3_): δ = −57.48. HRMS [ESI]^+^ calculated for C_10_H_5_F_3_O_3_: 230.0191; found [M]^+^ 230.0190.

### 8.3. General Procedure for the Synthesis of Various Alkoxyl Courmarin Derivatives

Varied alkoxy-substituted coumarins were prepared by using the corresponding alkyl bromide. The 8-hydroxycoumarin or 6,7-dihydroxycoumarin, alkyl bromide and potassium carbonate were mixed in DMF. The reaction mixture was heated to 200 °C for 1–2 h and then cooled to room temperature. The reaction mixture was diluted with H_2_O and extracted with dichloromethane. The organic layers were combined, dried over anhydrous MgSO_4_ and concentrated under vacuum. The residue was purified by silica gel column chromatography (*n*-hexane/rthyl acetate (7:3)) to give the various alkoxy-substituted coumarins.

*8-n-Butoxycoumarin* (**31**). Yield: 22.2%. White needle-like crystals. M.P.: 79–81°C. ^1^H-NMR (400 MHz, CDCl_3_): δ 7.65 (1H, d, *J* = 9.6), 7.17–7.13 (1H, m), 7.06–7.00 (2H, m), 6.40 (1H, d, *J* = 9.6 Hz), 4.09–4.06 (2H, m), 1.86–1.79 (2H, m), 1.54–1.52 (2H, m), 0.98–0.94 (3H, m). ^13^C- NMR (100 MHz, CDCl_3_): δ 160.4, 146.8, 143.9, 143.6, 124.2, 119.5, 119.1, 116.7, 115.0, 69.1, 31.1, 19.1, 13.7; HRMS [ESI]^+^ calculated for C_13_H_15_O_3_ [M + H]^+^ 218.0943; found 218.0944.

*6,7-Dialloxycoumarin* (**44**). Yield: 74.6%. White needle-like crystals. M.P.: 84–85 °C. ^1^H-NMR (400 MHz, CDCl_3_): δ 7.56 (1H, d, *J* = 9.6 Hz), 6.86 (1H, s), 6.82 (1H, s), 6.24 (1H, d, *J* = 9.6 Hz), 6.10–5.99 (2H, m), 5.46–5.28 (4H, m), 4.65–4.59 (4H, m). ^13^C-NMR (100 MHz, CDCl_3_): δ 161.3, 152.3, 149.9, 145.3, 143.2, 132.7, 131.9, 118.5, 118.0, 113.4, 111.5, 110.9, 101.5, 70.4, 69.8; HRMS [ESI]^+^ calculated for C_15_H_14_O_4_: 258.0892; found [M + H]^+^ 259.0893.

*6,7-Di-n-butoxycoumarin* (**46**). Yield: 89.9%. Yellow needle-like crystals. M.P.: 77–79 °C. ^1^H- NMR (400 MHz, CDCl_3_): δ 7.56 (1H, d, *J* = 9.6 Hz), 6.84 (1H, s), 6.78 (1H, s), 6.22 (1H, d, *J* = 9.6 Hz), 4.04–3.96 (4H, m), 1.85–1.75 (4H, m), 1.51–1.46 (4H, m), 0.98–0.96 (6H, m). ^13^C-NMR (100 MHz, CDCl_3_): δ 161.5, 153.1, 150.0, 146.6, 143.3, 113.1, 111.2, 110.5, 110.9, 69.6, 68.9, 31.1, 30.7, 19.1, 19.1, 13.7, 13.7; HRMS [ESI]^+^ calculated for C_17_H_22_O_4_: 290.1518; found [M]^+^ 290.1519.

*6,7-Dipentoxycoumarin* (**47**). Yield: 48.6%. Yellow solid. M.P.: 57–59 °C. ^1^H-NMR (400 MHz, CDCl_3_): δ 7.56 (1H, d, *J* = 9.6 Hz), 6.83 (1H, s), 6.78 (1H, s), 6.23 (1H, d, *J* = 9.6 Hz), 4.03–3.96 (4H, m), 1.88–1.78 (4H, m), 1.46–1.35 (4H, m), 0.93–0.89 (6H, m). ^13^C-NMR (100 MHz, CDCl_3_): δ 161.5, 153.1, 150.0, 146.0, 143.3, 113.1, 111.2, 110.4, 100.9, 69.8, 69.2, 28.8, 28.4, 28.1, 28.0, 22.3, 22.3, 13.9, 13.9; HRMS [ESI]^+^ calculated for C_19_H_26_O_4_: 318.1831; found [M]^+^ 318.1834.

*6,7-Di-isopentoxycoumarin* (**48**). Yield: 84.2%. White solid. ^1^H-NMR (400 MHz, CDCl_3_): δ 7.57 (1H, d, *J* = 9.6 Hz), 6.84 (1H, s), 6.79 (1H, s), 6.23(1H, d, *J* = 9.2 Hz), 4.07–3.99 (4H, m), 1.87–1.80 (2H, m), 1.76–1.55 (4H, m), 0.96–0.94 (12H, m). ^13^C-NMR (100 MHz, CDCl_3_): δ 161.5, 153.1, 150.0, 146.0, 143.3, 113.1, 111.2, 110.4, 100.9, 68.3, 67.7, 37.8, 37.4, 25.1, 22.5, 22.5; HRMS [ESI]^+^ calculated for C_19_H_26_O_4_: 318.1831; found [M]^+^ 318.1832.

*6,7-Dihexoxycoumarin* (**49**). Yield: 51.0%. Yellow solid. ^1^H-NMR (400 MHz, CDCl_3_): δ 7.56 (1H, d, *J* = 9.5 Hz), 6.83 (1H, s), 6.78 (1H, s), 6.22 (1H, d, *J* = 9.5 Hz), 4.03–3.96 (4H, m), 1.86–1.77 (4H, m), 1.46–1.32 (12H, m), 0.89–0.87 (6H, m). ^13^C-NMR (100 MHz, CDCl_3_): δ 161.5, 153.1, 150.0, 146.0, 143.3, 113.0, 111.2, 110.4, 100.8, 69.8, 69.2, 31.4, 31.4, 29.8, 28.7, 25.6, 25.5, 22.5 (2C), 13.9 (2C); HRMS [ESI]^+^ calculated for C_21_H_31_O_4_ [M + H]^+^347.2215; found [M]^+^ 346.4605.

*6,7-Digeranoxycoumarin* (**51**). Yield: 55.2%. Yellow solid. ^1^H-NMR (400MHz, CDCl_3_): δ 7.54 (1H, d, *J* = 9.6 Hz), 6.82 (1H, s), 6.75 (1H, s), 6.19 (1H, d, *J* = 9.2 Hz), 5.46–5.40 (2H, m), 5.01 (2H, s), 4.46–4.51 (4H, m), 2.89 (2H, s), 2.82 (2H, s), 2.06–1.91 (8H, m), 1.73–1.53 (18H, m). ^13^C-NMR (100 MHz, CDCl_3_): δ = 161.5, 152.2, 150.0, 145.6, 143.3, 141.5, 141.2, 131.8, 131.8, 123.6, 123.5 119.3, 118.8, 113.1, 111.2, 110.8, 101.3, 66.6, 66.3, 39.4, 39.4, 26.2, 26.1, 25.6, 25.5, 17.4 (2C), 16.7, 16.6; HRMS [ESI]^+^ calculated for C_29_H_38_O_4_: 450.2770; found 450.2772.

### 8.4. Cell Lines

Human hepatocellular carcinoma cell line (Hep 3B) was purchased from the Bioresource Collection and Research Center (Hsinchu, Taiwan).

### 8.5. Cell Culture

Cells were maintained in minimum essential medium (MEM, Invitrogen) containing 10% fetal bovine serum (HyClone^®^, Tianjin, China), 100 units/mL penicillin, 100 μg/mL streptomycin, 2 mM L-glutamine (penicillin-streptomycin-glutamine 100X from GIBCO, Invitrogen) and 1 mM sodium pyruvate at 37 °C humidified incubator with 5% CO_2_.

### 8.6. Cell Cytotoxicity Assay of Coumarin Derivatives

The colorimetric assay for cellular growth and survivals described by Hansen et al. with modifications [57]. The MTT (3-(4,5-cimethylthiazol-2yl)-2,5-diphenyltetrazolium bromide, Sigma-Aldrich Inc., Germany) assay was utilized to determine the IC_50_ value of each compound. Cells (5000 cells/well) were seeded into 96-well plates for 24 h and subsequently the cells were treated with vehicle or tested compounds at pre-determined concentration. After 72 h treatment, MTT solution was added to the final concentration of 0.5 mg/mL and continue cultured for 2 h at 37 °C. Afterward, the cells were lysed with lysis buffer (40% DMF and 20% SDS in H_2_O) overnight at 37 °C to lysate the cells. The absorbance at 570 nm was then detected by a microplate reader and the IC_50_ value was calculated.

### 8.7. Reverse Transcription Polymerase Chain Reaction (RT-PCR)

Total RNA was isolated from control and treated cells using a TRIzol reagent (Invitrogen) according to the manufacturer’s instruction. The RNA concentration was measured by a spectrophotometer and equal amounts of total RNA (2 μg) were reverse—transcribed using a RevertAid First Strand cDNA Synthesis Kit (K1622, Fermentas, Hanover, MD, USA). Amplification of cDNA was performed in the PCR reaction buffer (0.2 mM dNTP, 1.5 mM MgCl_2_, and 0.5 μM of each primer) containing 2.5 units of *Taq* DNA polymerase. The PCR products were resolved by agarose electrophoresis, visualized by ethidium bromide straining and quantified by Image J^®^. The PCR cycling procedures and sequences of primers are listed below. PCR conditions: (1) UGT1A1: 94 °C, 30 s; 62 °C, 45 s; 72 °C, 30 s; 27 cycles; (2) MRP2: 94 °C, 30 s; 62 °C, 45 s; 72 °C, 30 s; 27 cycles; (3) CAR: 94 °C, 30 s; 59 °C, 45 s; 72 °C, 30 s; 35 cycles; (4) GADPH: 94 °C, 30 s; 62 °C, 45 s; 72 °C, 30 s; 27 cycles; (4) gtPBREM: 94 °C, 30 s; 63 °C, 30 s; 68 °C, 60 s; 30 cycles.

### 8.8. Primer Sequences


**Primer**

**Sequence**
UGT1A1Forward: 5′-TGCAAAGCGCATGGAGACTA-3′
Reverse: 5′-GAGGCGCATGATGTTCTCCT-3′MRP2Forward: 5′-AGGTGAGGATTGACACCAACCA-3′
Reverse: 5′-AGGCAGTTTGTGAGGGATGACT-3′CARForward: 5′-GTGCTGCCTCTGGTCACACACT-3′
Reverse: 5′-GAGGCCCGCAGAGGAAGTTT-3′GAPDHForward: 5′-GTCTCCTCTGACTTCAACAGCG-3′
Reverse: 5′-ACCACCCTGTTGCTGTAGCCAA-3′gtPBREMForward: 5′-GTTTCCGCTAGCTACACTAGTAAAGGTCACTC-3′
Reverse: 5′-GTTTAACTCGAGCCCTCTAGCCATTCTGGATC-3′

### 8.9. Cloning and Transfection

The genomic DNA was isolated from Hep3B cells using Wizard^®^ Genomic DNA Purification Kit (Promega, Fitchburg, WI, USA). The promoter region of *UGT1A1* gene, gtPBREM element, was amplified by AccuPrime^TM^ Taq DNA Polymerase High Fidelity Kit (Invitrogen, MA, USA). The sequence of the amplified element was confirmed by DNA sequencing company and cloned into pGL4 luciferase reporter vector. The pGL4-gtPBREM plasmid was transfected into Hep3B cells. The stable pGL4-gtPBREM transfected Hep3B cells, Hep3B-gtPBREM cells, were established after G418 selection. The luciferase activity represented of CAR activation ability was measured using individual established clone. Further, CAR-shRNA was obtained from the Genomic Research Center and transfected into Hep3B-gtPBREM cells. The transient transfected shCAR-Hep3B-gtPBREM cells were used for further luciferase assays.

### 8.10. Luciferase Assays

The Hep3B-gtPBREM cells and shCAR-Hep3B-gtPBREM cells (1.5 × 10^5^ cells/well) were seeded into 12-well plates for overnight then pre-determined concentration compounds were added for desired time. After treatment, transfected cells were washed twice in PBS, lysated with 1× passive lysis buffer and luciferase intensity of equal amount of cell lysate was measured by a Synergy HT Multi-Mode Microplate Reader (BioTek, Winooski, VT, USA).

### 8.11. Nuclear and Cytoplasmic Extraction

Cells were harvest by centrifugation after trypsin-EDTA treatment. The ice-cold CER I (10 μL/1 × 10^6^ cells) was added to the cell pellet following the manufacturer’s instruction. The tube was vigorously vortexed on the highest setting for 15 s to fully suspend the cell pellet, incubated on ice for 10 min, then ice-cold CER II was added to the tube and vortexed for 5 s on the highest setting. After incubating in ice for 1 min, tube was vortexed for 5 s on the highest setting then subjected to centrifuge for 5 min at 16,000× *g*. The supernatant (cytoplasmic fraction) was transfected to a clean pre-chilled tube and the nuclear fraction (pellet) was re-suspended in ice-cold NER. After 40 min incubation on ice, the nuclear fraction was subjected to centrifuge for 10 min at 16,000× *g* and the supernatant was transferred to a pre-chilled tube for further investigation.

### 8.12. Protein Extraction

1 × 10^6^ cells were seeded in a 10 cm plate, incubated at 37 °C for overnight and treated with various concentrations of compounds for the indicated times. After incubation, the culture medium was removed and washed twice with 2 mL ice-cold 1× PBS. Three hundred μL of protein lysis buffer (150 mM NaCl, 50 mM Tris-HCl (pH 7.4), 1% SDS, 1% Triton X-100, 1 mM PMSF) was added into each plate, scraped with a rubber policeman, collected into a premarked microtube and the incubated in ice for 1 h, sonicated by Ultrasonic Cell Disruptor for three time at 5 s each. The cell lysated was cleared by centrifugation at 12,000× *g* for 15 min at 4 °C. Supernatants were transferred into a fresh marked microtube and stored at −20 °C for future use.

### 8.13. Western Blots

Protein concentration of each sample was determined using a BCA Protein Assay Kit (Pirece^®^, Thermo Scientific). Different percentages of SDS-PAGE gels were prepared according to the molecular size of proteins of interest. After complete polymerization of stacking gels, each sample with equal amount of total protein was separated in polyacrylamide gels (stacking gel: 50 v, 60 min; separating gel: 100 v, 60 min). After separating, wet-transfer method was utilized to transfer proteins onto the PVDF membrane. The gels were assembled in the transfer sandwich and put in a transfer tank filled with transfer buffer (25 mM glycine, 0.15% ethanolamine, 20% methanol). The electro-transfer was performed at the voltage of 100 volt for 80 min at 4 °C. After transfer, PVDF-membrane was blocked with 5% BSA in 1× TBS-Tween at room temperature for 1 h. Membrane was washed with 10 mL 1× TBS-Tween three times and incubated with primary antibodies overnight at 4 °C with gentle agitation. After primary antibody was removed, the membrane was washed three times with 10 mL 1× TBS-Tween for 10 min at room temperature with constant shaking. The horseradish peroxide (HRP)-conjugated secondary antibody was added to react with the membrane for 1 h at room temperature with gentle agitation. After incubation, membrane was washed with 1× TBS-Tween 3 times for 10 min. For detection, the membrane was immersed completely in the mixture of 0.5 mL luminosol reagent and 0.5 mL peroxidase for 1 min. The emitted fluorescence from the membrane was caught on a LAS-4000 biomolecule imager (FUJI.).

### 8.14. Immunofluorescence

Hep3B cells (4 × 10^5^) were grown on coverslips for overnight then washed twice with 1× PBS and fixed with 4% paraformaldehyde in 1× PBS for 30 min at room temperature. Cells were then permeabilized with 1× PBS containing 0.1% (*v/v*) Triton X-100 for 10–15 min and blocked in 1× PBS containing 5% BSA for 1 h at room temperature. CAR antibody (1:200 dilutions) was added and reacted at room temperature for 2 h in 1× PBS containing 5% BSA. FITC-conjugated goat anti-rabbit IgG antibody was added for another 1 h at room temperature. Coverslips were mounted and images were acquired under a fluorescence microscope.

### 8.15. Animals

Six-week old male C57/BL6 mice (*n* = 6) were acquired from the Animal Center of the College of Medicine, National Taiwan University (Taipei, Taiwan) and 6-week-old male *db/db* mice (BKS.Cg-Dock7m +/+ Leprdb/JNarl) (*n* = 12) were acquired from the National Laboratory Animal Center (Tainan, Taiwan). Experimental animals were allowed to acclimate to the controlled photoperiod (a cycle of 12-h light/12-h dark), humidity (40–60% relative humidity) and temperature (22 ± 2 °C) with ad libitum supply of standard chow diets and drinking water for one week prior to the experimental treatment in accordance with the Guide for the Care and Use of Laboratory Animals (Institute of Laboratory Animal Resources Commission on Life Sciences, 2011) [58]. Acclimated animals were randomly separated into three cohorts: control group (C57/BL6 mice treated with vehicle for two weeks, *n* = 6), db/db group (*db/db* mice treated with vehicle for two weeks, *n* = 6), and db/db+SP group (*db/db* mice treated with 100 mg/kg scoparone for two weeks, *n* = 6). (IACUC Approval NO.: 20140532; Duration: 2015.01.01–2015.12.31; Ethical Committee: National Taiwan University College of Medicine and College of Public Health Institutional Animal Care and Use Committee (IACUC).)

### 8.16. Oral Glucose Tolerance Test (OGTT) 

The control mice or diabetic mice with or without drugs treatment for 2 weeks received an oral glucose challenge (2 g/kg). Mice were under slight anesthesia by an intraperitoneal injection of pentobarbital (50 mg/kg) and blood samples (20–50 μL via orbital sinus at each time point) were collected following: 0, 15, 30, 60, 90, and 120 min after delivery of the glucose load. Blood glucose levels were determined using SURESTEP blood glucose meter (Lifescan). Glucose tolerance was determined and performed as the curve (AUC) and delta AUC (ΔAUC) using Prism 5 software (GraphPad).

### 8.17. Determination of Blood Insulin and Fructosamine

To determine the amount of insulin and fructosamine after fasting overnight, blood samples (0.5–0.8 mL) of euthanasia animals were collected. After centrifugation, the serum was analyzed by insulin and fructosamine immunoassay kits according to the respective instructions of the manufacturer (Mercodia AB Inc., Uppsala, Sweden; Hospitex Diagnostics Lp, League City, TX, USA). The homeostasis model assessment of insulin resistance (HOMA-IR) index is calculated with the formula of the values of fasting glucose and insulin divided by 22.5 as previously described [59].

### 8.18. Statistical Analysis

The values in the graphs are given as mean ± S.E.M. The significance of difference was evaluated by the paired Student’s t-test. When more than one group was compared with one control, significance was evaluated according to one-way analysis of variance (ANOVA). Probability values of <0.05 were considered to be significant.

## Figures and Tables

**Table 1:** Mono-substituted coumarin structures and their CAR activation.

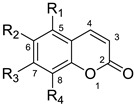						
**Compound**	**R_1_**	**R_2_**	**R_3_**	**R_4_**	**CAR Activation Fold ^a^**	**Ref**
**1** **5-hydroxycoumarin**	OH	H	H	H	6% ± 22%	[25]
**2** **5-methoxycoumarin**	*O*-methyl	H	H	H	−20% ± 6%	[26,27]
**3**	*O*-acetyl	H	H	H	−32% ± 12%	[28]
**4**	*O*-allyl	H	H	H	−15% ± 6%	[29]
**5**	*O*-*n*-butyl	H	H	H	−9% ± 14%	[29]
**6**	*O*-prenyl	H	H	H	−5% ± 9%	[30]
**7**	*O*-geranyl	H	H	H	−18% ± 11%	[30]
**8**	*O*-farnasyl	H	H	H	2% ± 14%	[30]
**9** **6-hydroxycoumarin**	H	OH	H	H	−8% ± 11 %	[31]
**10** **6-methoxycoumarin**	H	*O*-methyl	H	H	32% ± 5%	[32]
**11**	H	*O*-trifluoromethyl	H	H	−34% ± 1%	New
**12**	H	*O*-acetyl	H	H	−22% ± 3%	[33]
**13**	H	*O*-allyl	H	H	−17% ± 6%	[34]
**14**	H	*O*-n-butyl	H	H	37% ± 6%	[35]
**15**	H	*O*-prenyl	H	H	22% ± 2%	[30]
**16**	H	*O*-geranyl	H	H	18% ± 7%	[30]
**17**	H	*O*-farnasyl	H	H	−41% ± 9%	[30]
**18** **umbelliferone**	H	H	OH	H	−1% ± 18%	[31,36]
**19** **7-methoxycoumarin**	H	H	*O*-methyl	H	−9% ± 6%	[25,27,36]
**20**	H	H	*O*-trifluoromethyl	H	18% ± 2%	New
**21**	H	H	*O*-acetyl	H	−37% ± 12%	[37]
**22**	H	H	*O*-allyl	H	−46% ± 11%	[37]
**23**	H	H	*O*-n-butyl	H	−51% ± 7%	[38]
**24**	H	H	*O*-prenyl	H	−35% ± 13%	[30,39]
**25** **aurapten**	H	H	*O*-geranyl	H	−13% ± 4%	[30]
**26**	H	H	*O*-farnasyl	H	−29% ± 10%	[30]
**27** **8-hydroxycoumarin**	H	H	H	OH	40% ± 38%	[40]
**28** **8-methoxycoumarin**	H	H	H	*O*-methyl	−8% ± 10%	[41]
**29**	H	H	H	*O*-acetyl	−33% ± 13%	[28]
**30**	H	H	H	*O*-allyl	−34% ± 12%	[42]
**31**	H	H	H	*O*-n-butyl	4% ± 9%	[43]
**32**	H	H	H	*O*-prenyl	2% ± 23%	[30]
**33**	H	H	H	*O*-geranyl	−30% ± 4%	[30]
**34**	H	H	H	*O*-farnasyl	19% ± 25%	[30]

^a^ CAR activation fold was calculated: (the luminescence value of the tested compound- the luminescence value of scoparone)/the luminescence value of scoparone (as positive control) × 100%.

**Table 2:** Di-substituted coumarins and their CAR activation.

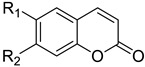				
**Compound**	**R_1_**	**R_2_**	**CAR Activation Fold ^a^**	**Ref**
**35** **esculetin**	OH	OH	−22% ± 12%	[36,44]
**36**	methyl	methyl	−17% ± 5%	[24]
**37** **isoscopoletin**	OH	*O*-methyl	−10% ± 6%	[45]
**38** **scopoletin**	*O*-methyl	OH	−1% ± 5%	[44]
**39** **scoparone**	*O*-methyl	*O*-methyl	-	[15,17]
**40** **ayapin**	-OCH_2_O-		7% ± 2%	[46]
**41**	-OCH_2_CH_2_O-		−50% ± 9%	[47]
**42**	*O*-ethyl	*O*-ethyl	−26% ± 8%	[37]
**43**	*O*-acetyl	*O*-acetyl	−11% ± 2%	[37]
**44**	*O*-allyl	*O*-allyl	−18% ± 15%	New
**45**	*O*-propyl	*O*-propyl	−37% ± 5%	[37]
**46**	*O*-*n*-butyl	*O*-*n*-butyl	−39% ± 13%	New
**47**	*O*-pentyl	*O*-pentyl	−10% ± 18%	New
**48**	*O*-isopentyl	*O*-isopentyl	−61% ± 2%	New
**49**	*O*-hexyl	*O*-hexyl	−5% ± 9%	New
**50**	*O*-prenyl	*O*-prenyl	50% ± 1%	[48,49,50]
**51**	*O*-geranyl	*O*-geranyl	−32% ± 11%	New
**52** **isoscopolin**	*O*-Glc	*O*-methyl	35% ± 5%	[17,51]
**53** **scopolin**	*O*-methyl	*O*-Glc	17% ± 26%	[17]

^a^ CAR activation fold was calculated: (the luminescence value of the tested compound- the luminescence value of scoparone)/the luminescence value of scoparone (as positive control) × 100%.

**Table 3:** Tri- and tetra-substituted coumarins and their CAR activation.

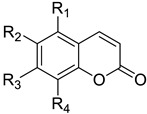						
**Compound**	**R_1_**	**R_2_**	**R_3_**	**R_4_**	**CAR Activation Fold ^a^**	**Ref**
**54**	-OCH_3_	-OCH_3_	-OCH_3_	H	−19% ± 3%	[39,44]
**55** **fraxinol**	-OCH_3_	-OH	-OCH_3_	H	−17% ± 2%	[39]
**56**	-OCH_3_	H	-OCH_3_	-OCH_3_	−14% ± 2%	[39]
**57** **leptodactylone**	-OCH_3_	H	-OCH_3_	-OH	2% ± 4%	[39]
**58**	H	-OCH_3_	-OCH_3_	-OCH_3_	0% ± 2%	[52]
**59**	H	-OCH_3_	-OCH_2_O-		36% ± 23%	[17,53]
**60**	-OCH_3_	-OCH_3_	-OCH_2_O-		12% ± 1%	[17,53]

^a^ CAR activation fold was calculated: (the luminescence value of the tested compound- the luminescence value of scoparone)/the luminescence value of scoparone (as positive control) × 100%.

## Data Availability

The data presented in this study are available on request from the corresponding author.

## Supplementary Materials

The online version of this article contains Supplementary Materials.
