# Supplementary material for: Studies of Coumarin Derivatives for Constitutive Androstane Receptor (CAR) Activation
Source: Molecules. 2020 Dec 31;26(1):164. doi: 10.3390/molecules26010164 (PMC7796031; doi:10.3390/molecules26010164)
Supplement: Supplementary file 1 [file molecules-26-00164-s001.pdf]

Supplement
Studies of Coumarin Derivatives for Constitutive Androstane Receptor (CAR) Activation

Shin-Hun Juang1, Min-Tsang Hsieh1,2, Pei-Ling Hsu1#, Ju-Ling Chen1,3#, Hui-Kang Liu4, Fong-Pin Liang1, Sheng-Chu Kuo1, Chen-Yuan Chiu5, Shing-Hwa Liu5, Chen-Hsi Chou3, Tian-Shung Wu3, Hsin-Yi Hung3,*

1 School of Pharmacy, China Medical University, Taichung 404, Taiwan

2 Chinese Medicine Research and Development Center, China Medical University Hospital, 2 Yude Road, Taichung 404, Taiwan

3 School of Pharmacy, College of Medicine, National Cheng Kung University, Tainan 701, Taiwan

4 National Research Institute of Chinese Medicine, Ministry of Health and Welfare, Taipei, 112, Taiwan.

5 Graduate Institute of Toxicology, College of Medicine, National Taiwan University, Taipei 100, Taiwan.

Content

S1 ^{1}H NMR (400 MHz, $CDCl_3$) spectrum of 6-trifluoromethoxylcoumarin (11) 3

S2 ^{13}C NMR (100 MHz, $CDCl_3$) spectrum of 6-trifluoromethoxylcoumarin (11) 4

S3 ^{19}F NMR (470 MHz, $CDCl_3$) spectrum of 6-trifluoromethoxylcoumarin (11) 5

S4 ^{1}H NMR (400 MHz, $CDCl_3$) spectrum of 7-trifluoromethoxylcoumarin (20) 6

S5 ^{13}C NMR (100 MHz, $CDCl_3$) spectrum of 7-trifluoromethoxylcoumarin (20) 7

S6 ^{19}F NMR (470 MHz, $CDCl_3$) spectrum of 7-trifluoromethoxylcoumarin (20) 8

S7 ^{1}H NMR (400 MHz, $CDCl_3$) spectrum of 8-*O*-*n*-Butylcoumarin (31) 9

S8 ^{13}C NMR (100 MHz, $CDCl_3$) spectrum of 8-*O*-*n*-Butylcoumarin (31) 10

S9 ^{1}H NMR (400 MHz, $CDCl_3$) spectrum of 6,7-Dialloxycoumarin (44) 11

S10 ^{13}C NMR (100 MHz, $CDCl_3$) spectrum of 6,7-Dialloxycoumarin (44) 12

S11 ^{1}H NMR (400 MHz, $CDCl_3$) spectrum of 6,7-Di-*n*-butoxycoumarin (46) 13

S12 ^{13}C NMR (100 MHz, $CDCl_3$) spectrum of 6,7-Di-*n*-butoxycoumarin (46) 14

S13 ^{1}H NMR (400 MHz, $CDCl_3$) spectrum of 6,7-Dipentoxycoumarin (47) 15

S14 ^{13}C NMR (100 MHz, $CDCl_3$) spectrum of 6,7-Dipentoxycoumarin (47) 16

S15 ^{1}H NMR (400 MHz, $CDCl_3$) spectrum of 6,7-Di-isopentoxycoumarin (48) 17

S16 ^{13}C NMR (100 MHz, $CDCl_3$) spectrum of 6,7-Di-isopentoxycoumarin (48) 18

S17 ^{1}H NMR (400 MHz, $CDCl_3$) spectrum of 6,7-Dihexoxycoumarin (49) 19

S18 ^{13}C NMR (100 MHz, $CDCl_3$) spectrum of 6,7-Dihexoxycoumarin (49) 20

S19 ^{1}H NMR (400 MHz, $CDCl_3$) spectrum of 6,7-Digeranoxycoumarin (51) 21

S20 ^{13}C NMR (100 MHz, $CDCl_3$) spectrum of 6,7-Digeranoxycoumarin (51) 22

S21 Effects of scoparone, 50 (PLH), and 6 on phosphoenopyruvate carboxykinse (PEPCK) mRNA expression induced by dexamethasone and 8-bromo-cAMP. 23

S22 Treatment of scoparone on db/db mice for two weeks improves glucose tolerance and insulin sensitivity. 25

S23 Preliminary pharmacokinetic studies of scoparone and 6,7-diprenoxycoumarin 28

S1 ^{1}H NMR (400 MHz, $CDCl_3$) spectrum of 6-trifluoromethoxylcoumarin (**11**)

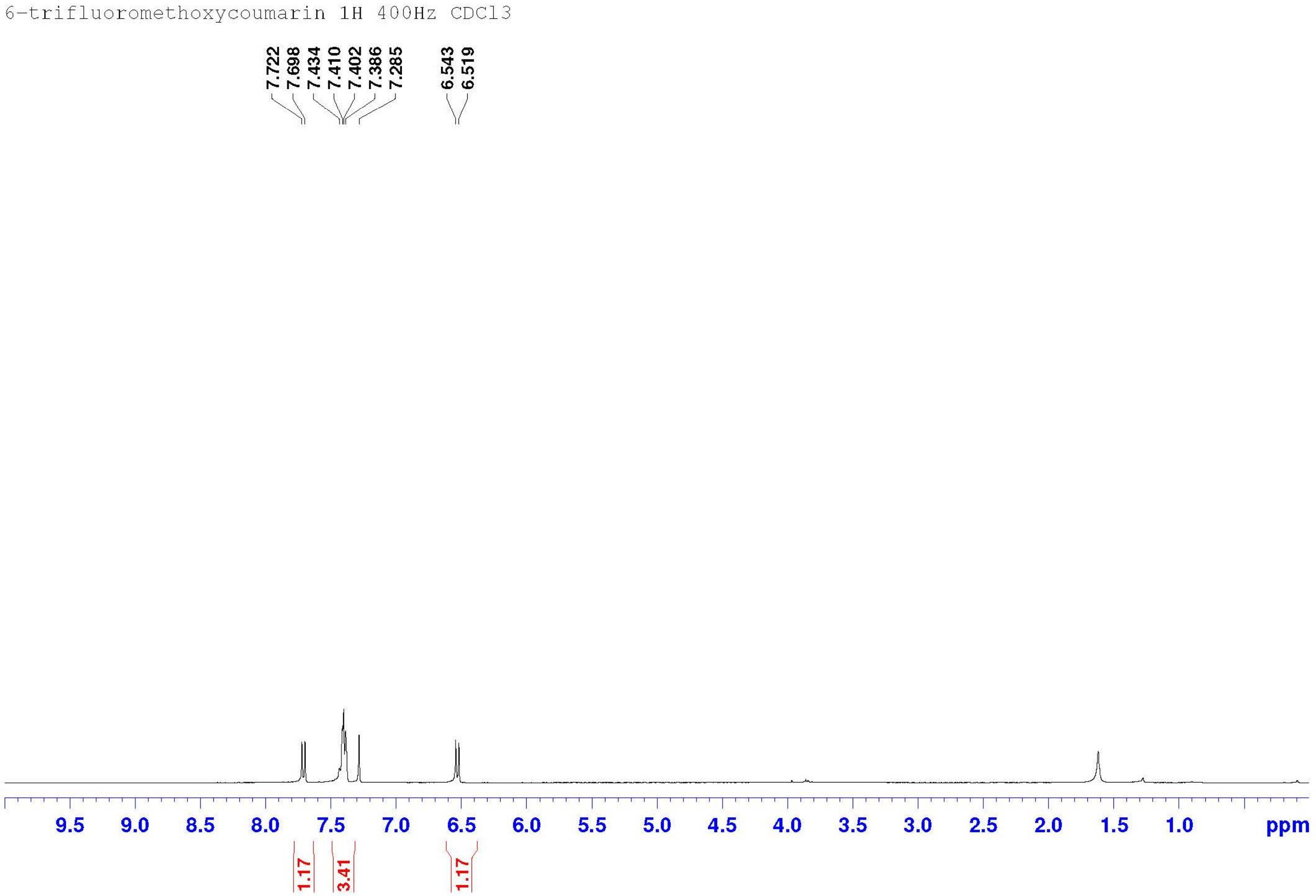

S2 ^{13}C NMR (100 MHz, $CDCl_3$) spectrum of 6-trifluoromethoxylcoumarin (**11**)

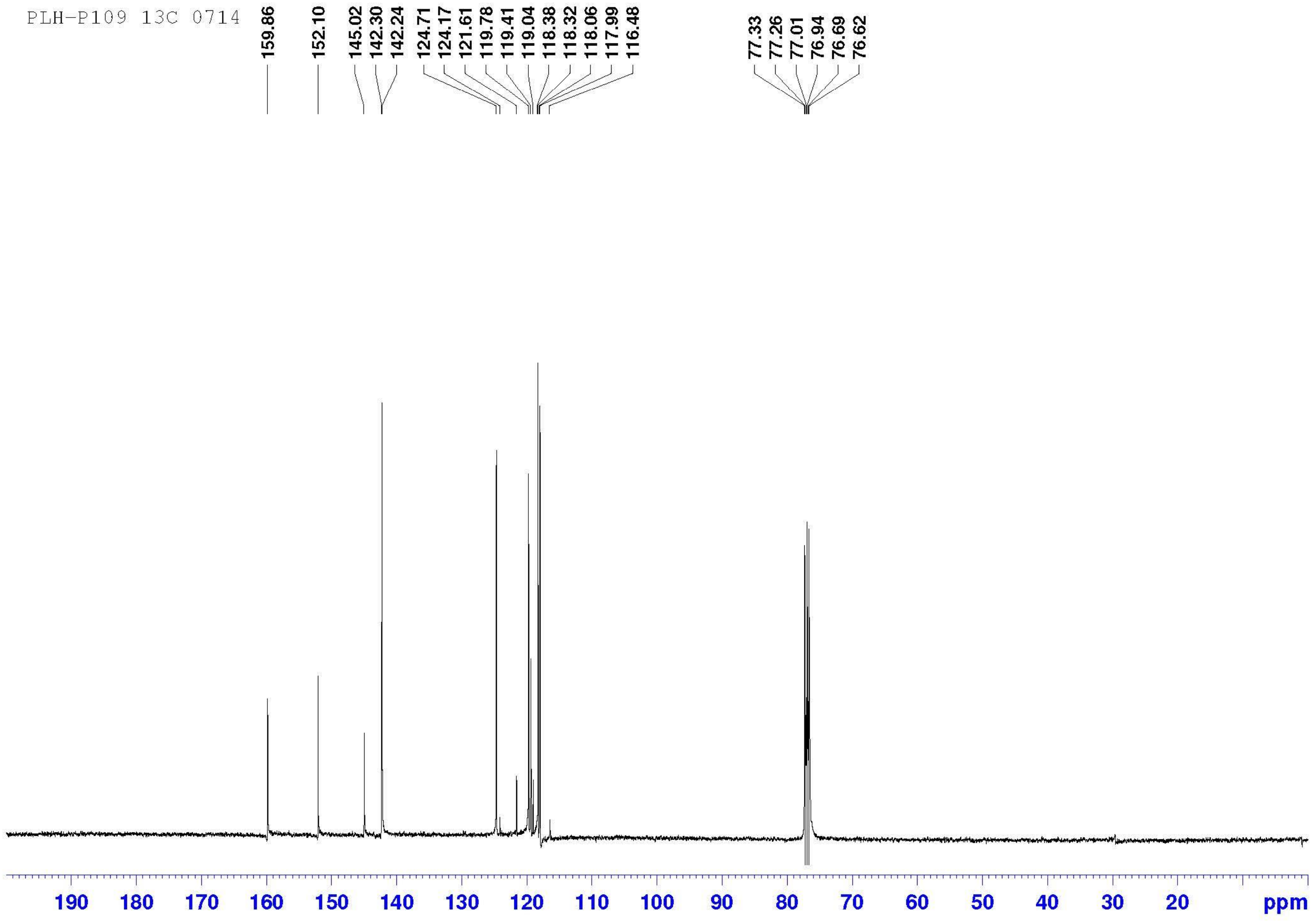

S3 ^{19}F NMR (470 MHz, $CDCl_3$) spectrum of 6-trifluoromethoxylcoumarin (**11**)

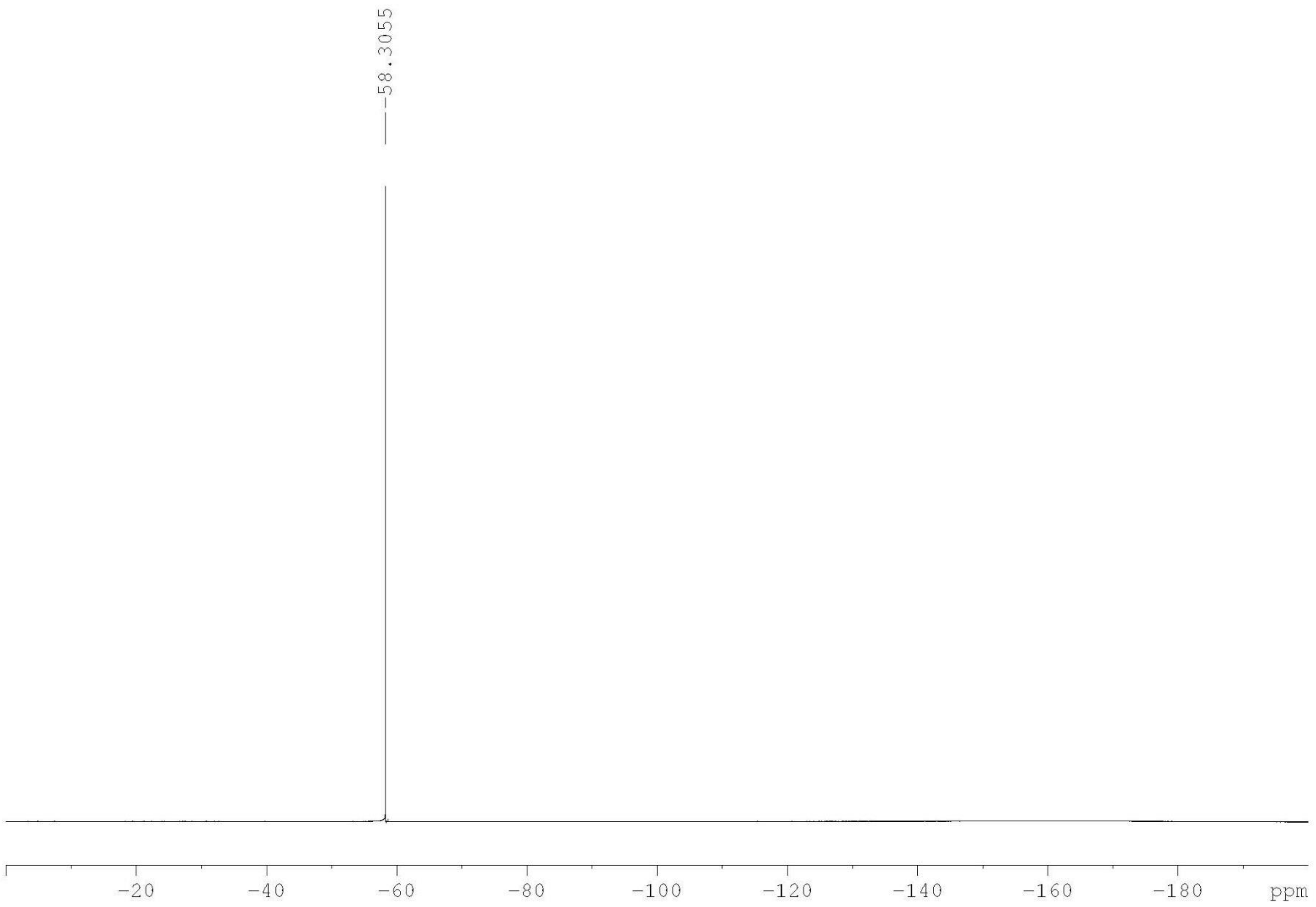

S4 ^{1}H NMR (400 MHz, $CDCl_3$) spectrum of 7-trifluoromethoxylcoumarin (**20**)

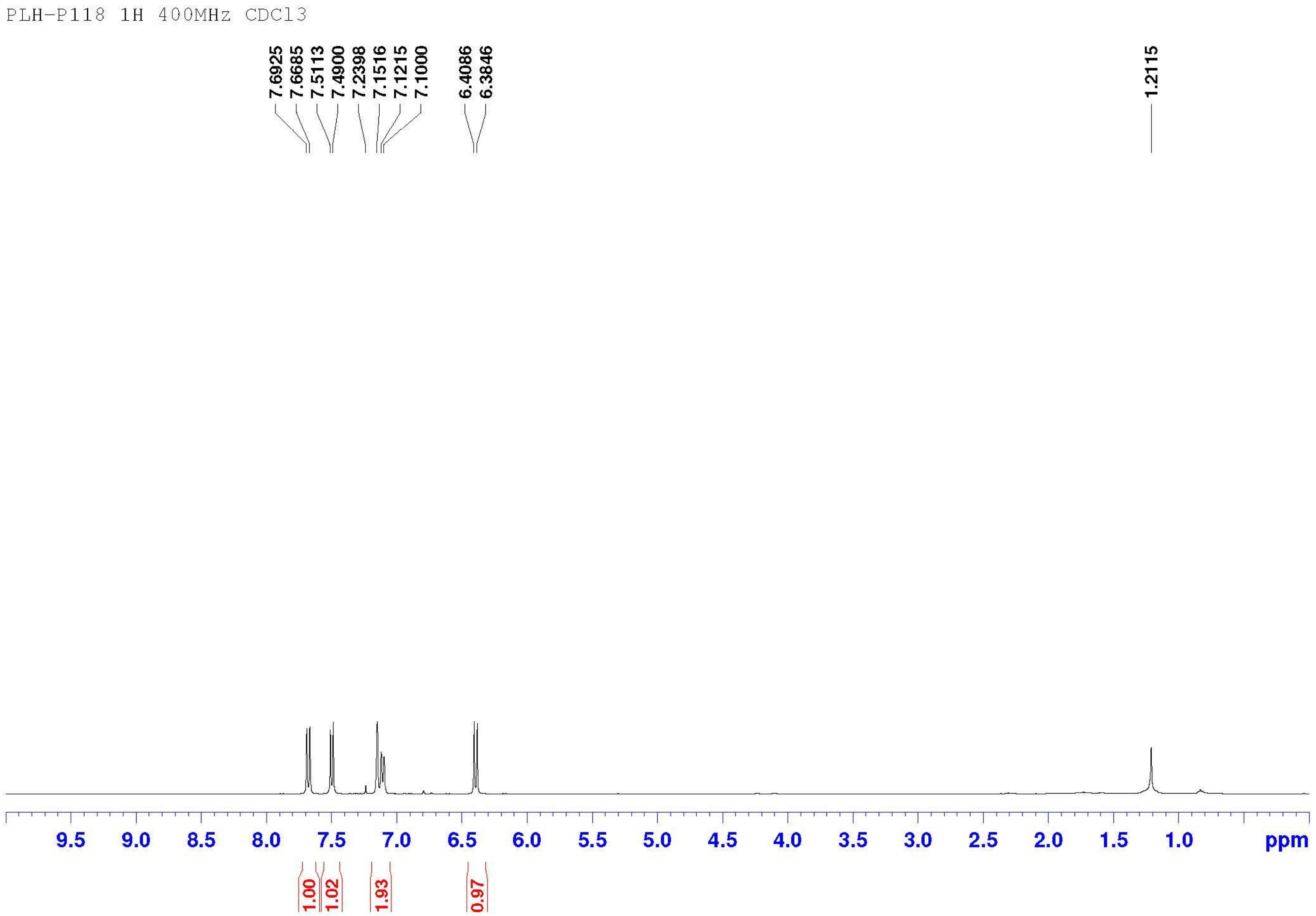

S5 ^{13}C NMR (100 MHz, CDCl$_3$) spectrum of 7-trifluoromethoxylcoumarin (**20**)

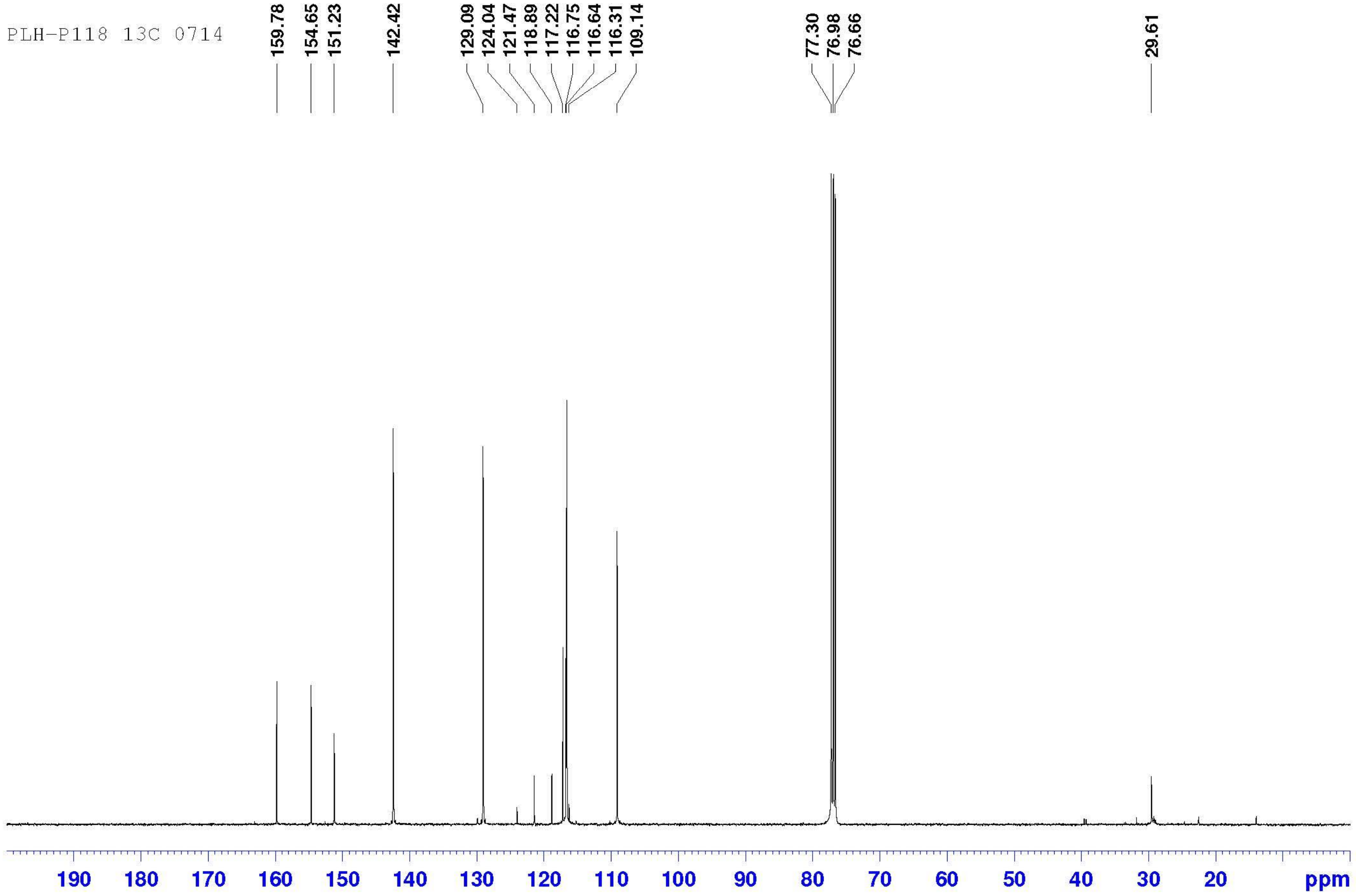

S6 ^{19}F NMR (470 MHz, $CDCl_3$) spectrum of 7-trifluoromethoxylcoumarin (**20**)

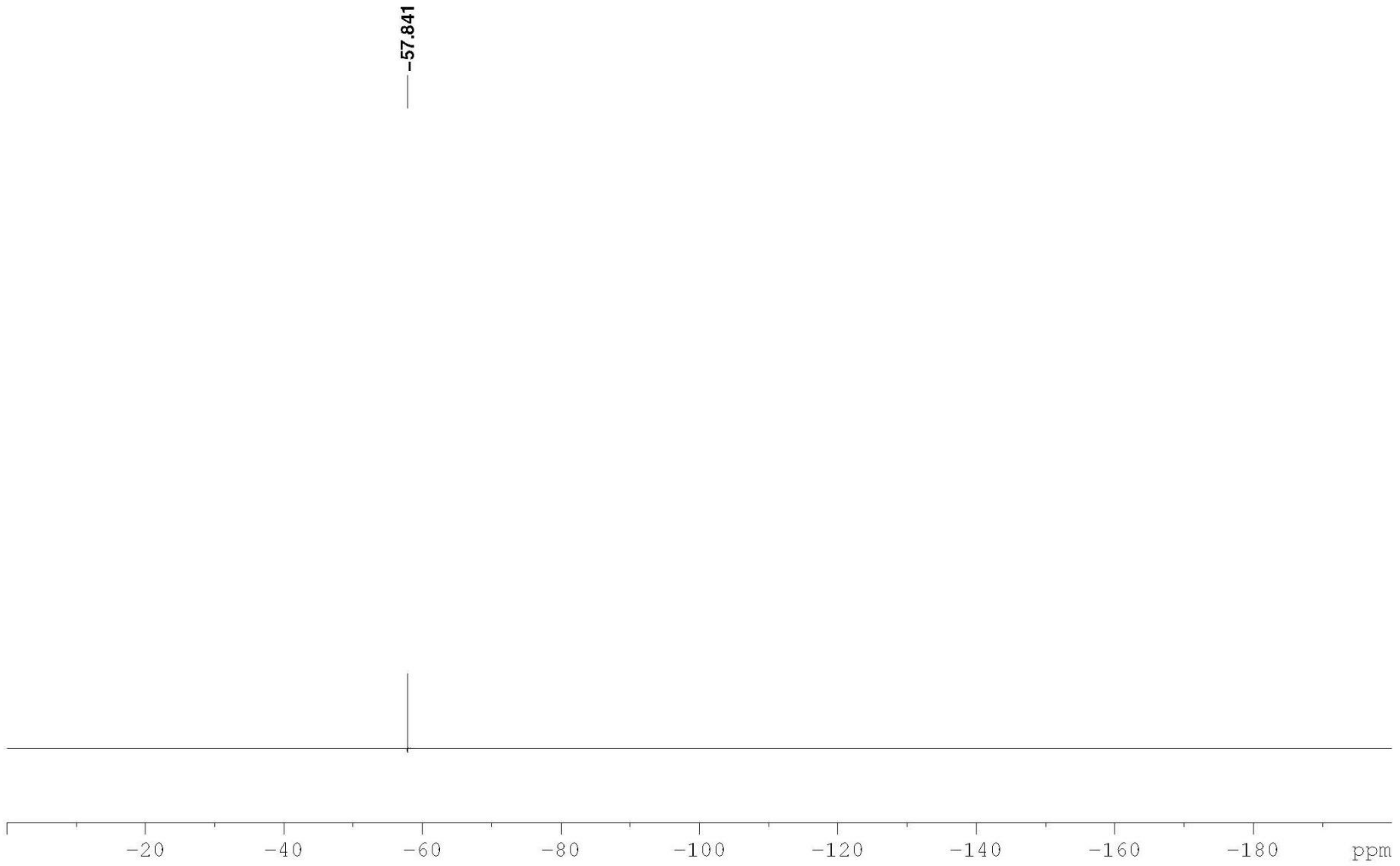

S7 ^{1}H NMR (400 MHz, $CDCl_3$) spectrum of 8-*O*-*n*-Butylcoumarin **(31)**

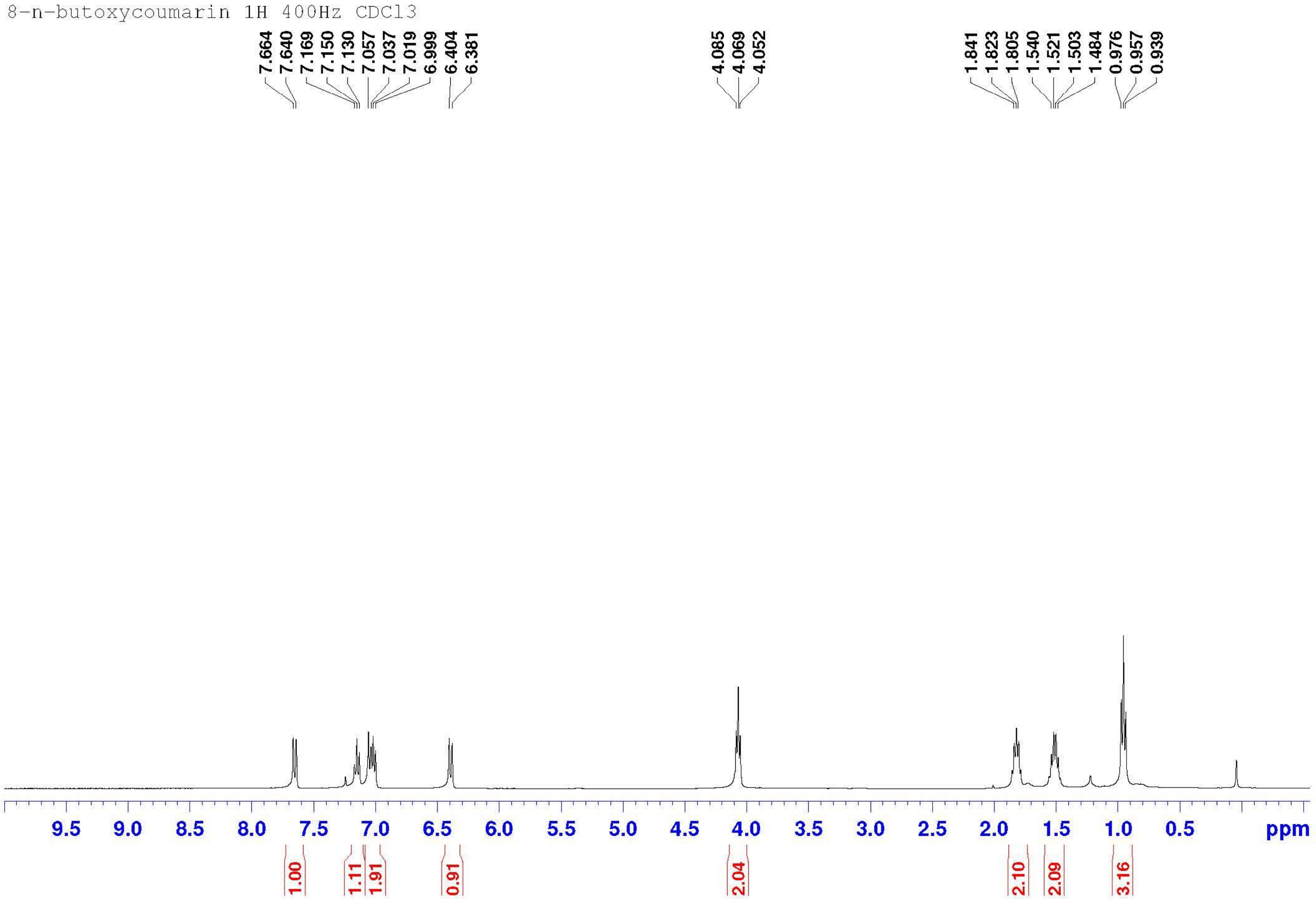

S8 ^{13}C NMR (100 MHz, $CDCl_3$) spectrum of 8-*O*-*n*-Butylcoumarin **(31)**

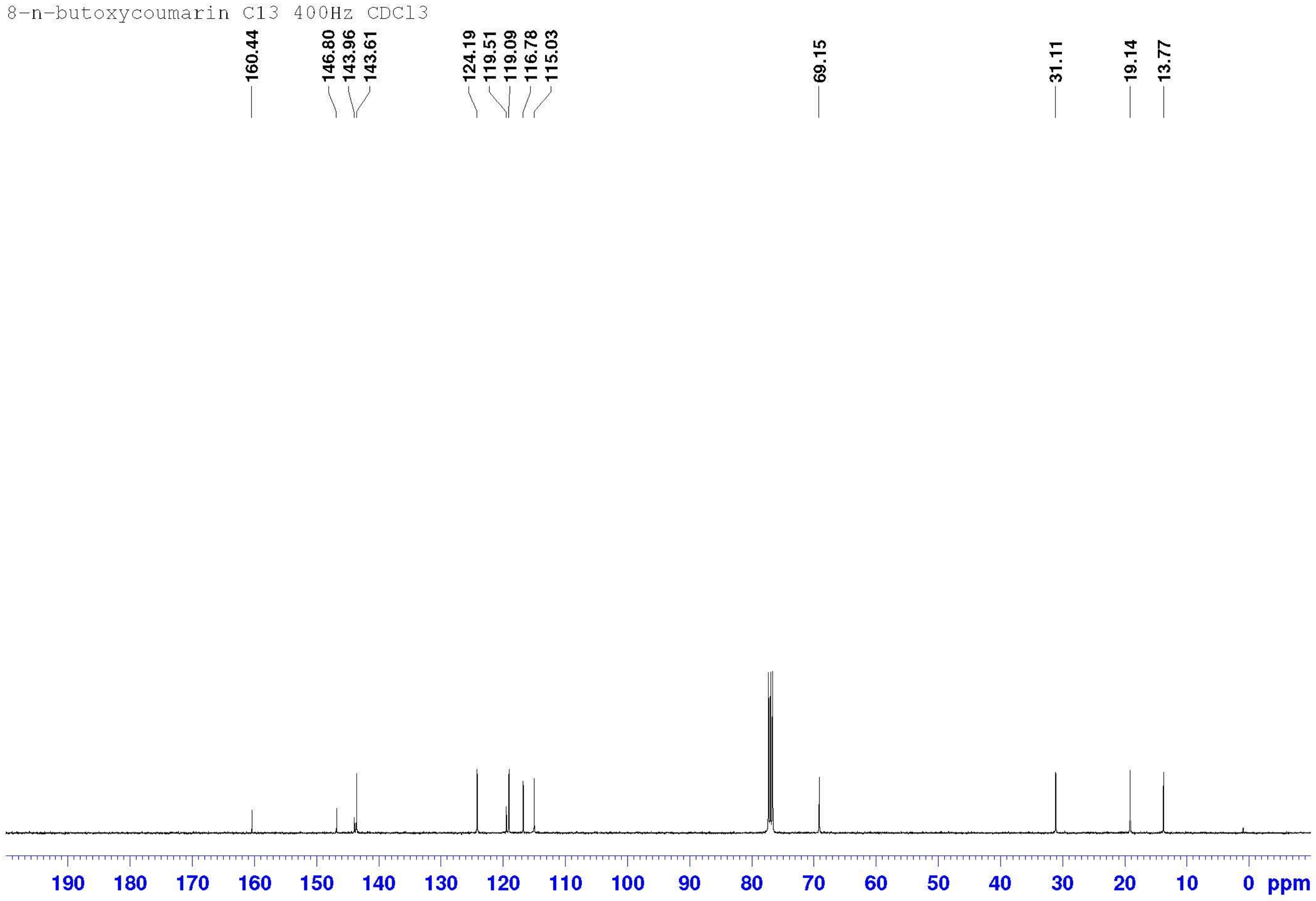

S9 ^{1}H NMR (400 MHz, $CDCl_3$) spectrum of 6,7-Dialloxycoumarin **(44)**

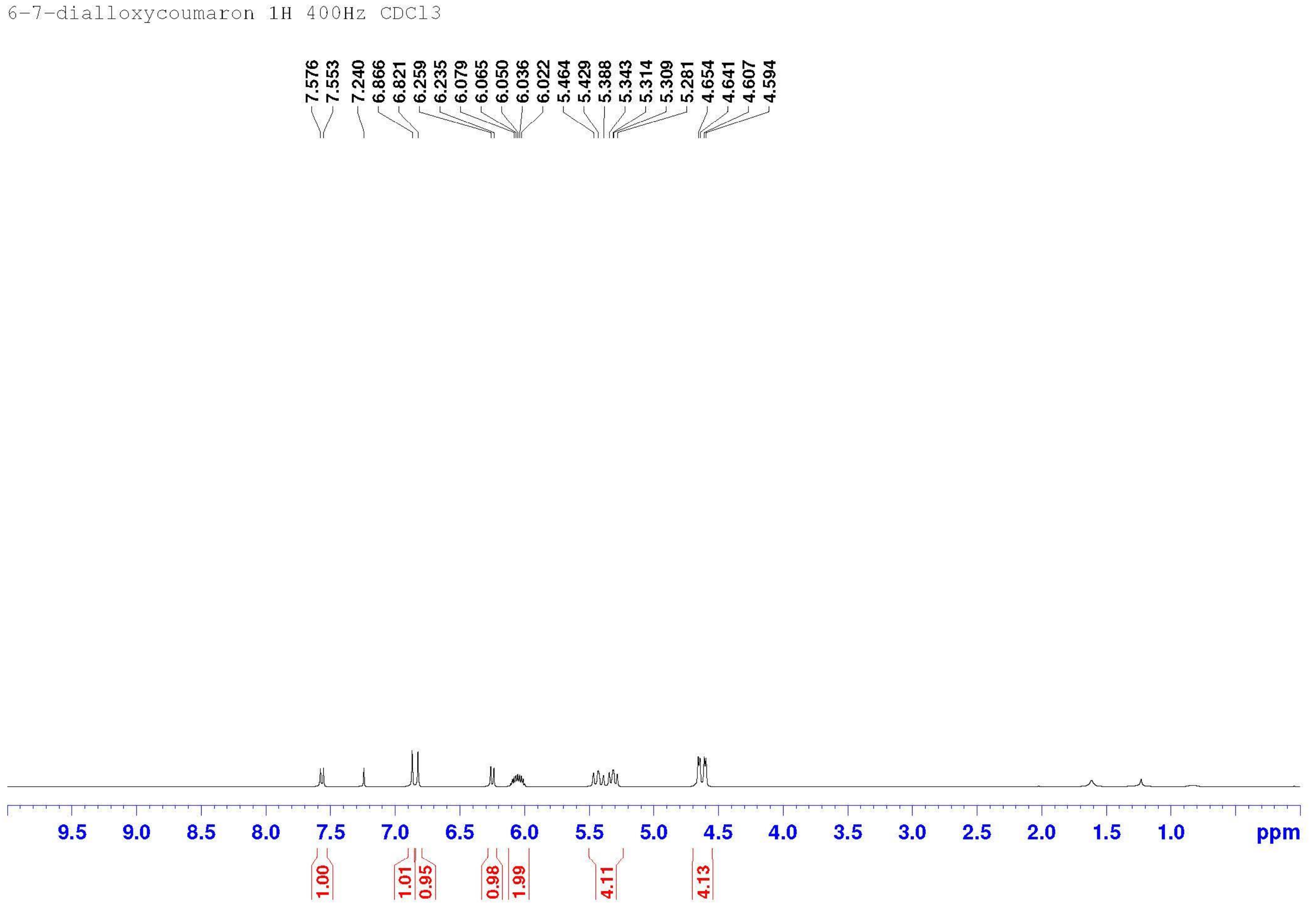

S10 ^{13}C NMR (100 MHz, $CDCl_3$) spectrum of 6,7-Dialloxycoumarin **(44)**

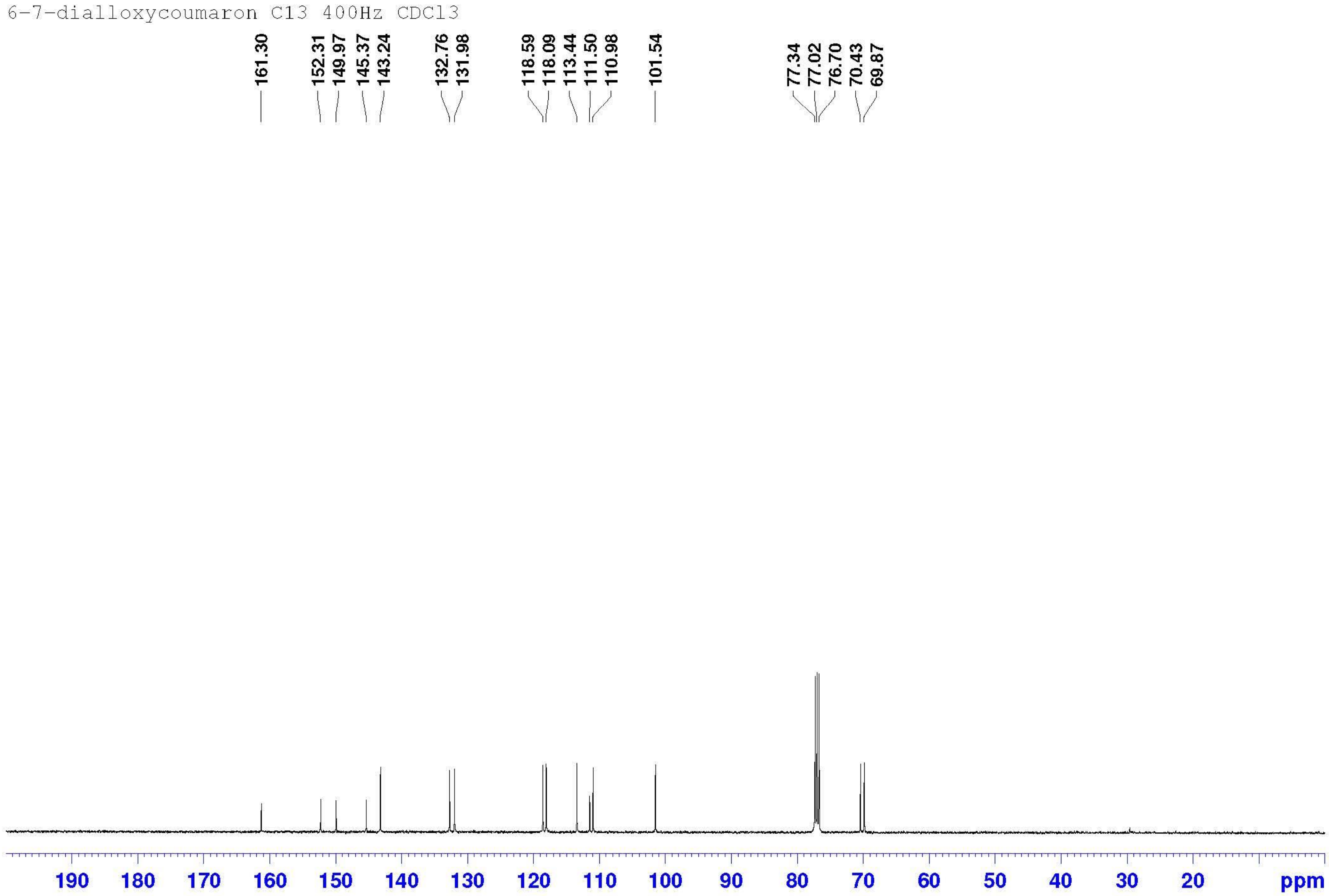

S11 ^{1}H NMR (400 MHz, $CDCl_3$) spectrum of 6,7-Di-*n*-butoxycoumarin **(46)**

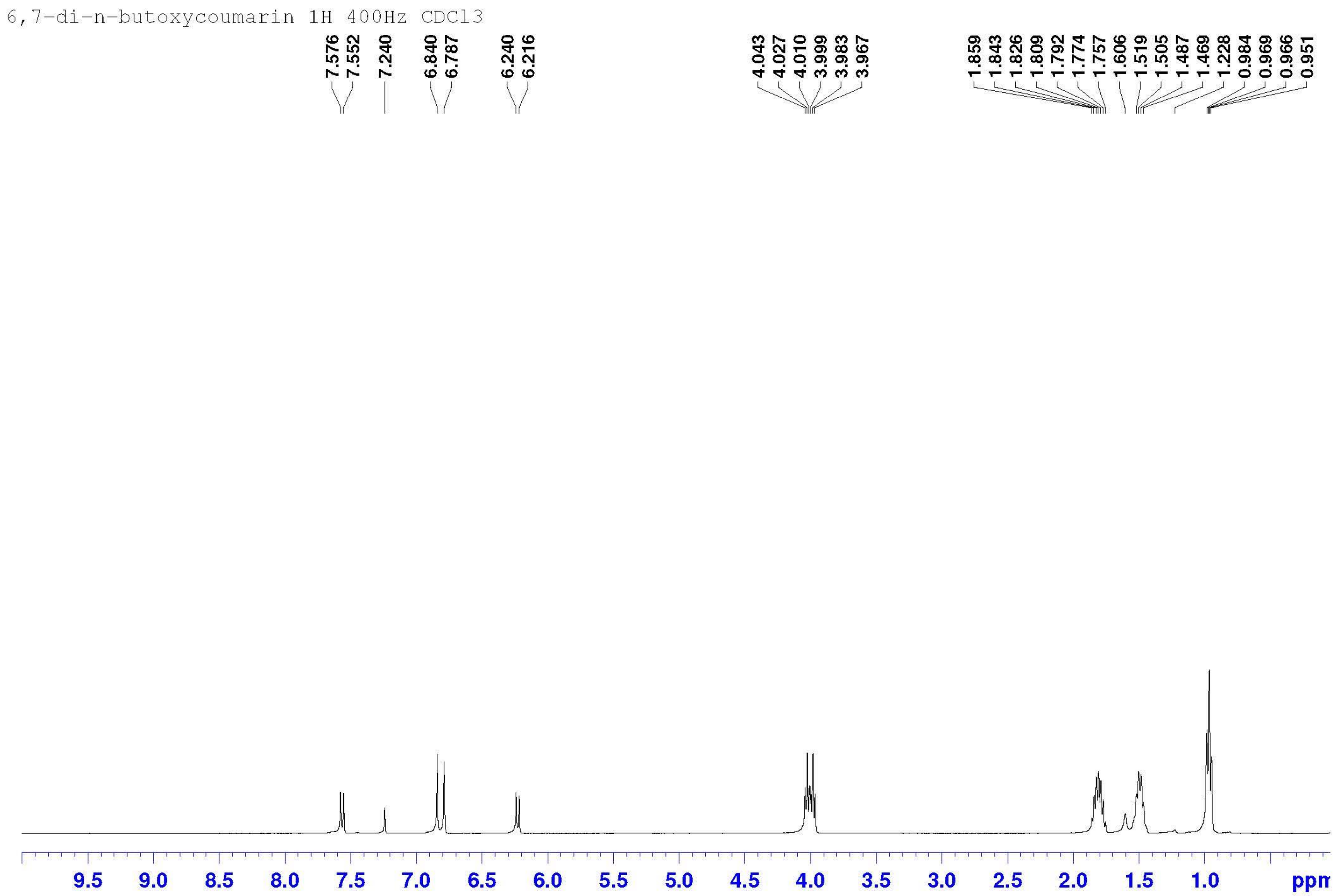

S12 ^{13}C NMR (100 MHz, $CDCl_3$) spectrum of 6,7-Di-*n*-butoxycoumarin **(46)**

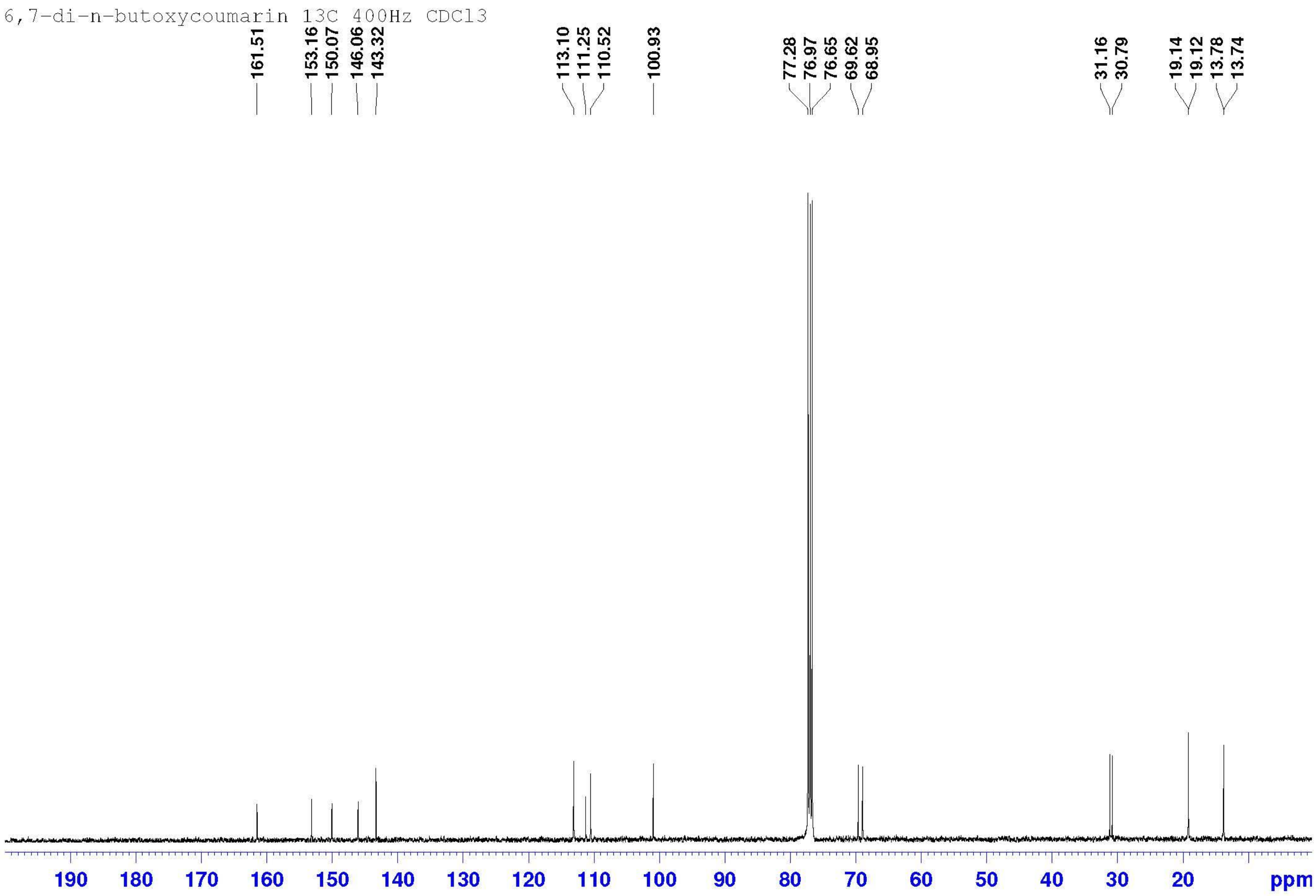

S13 ^{1}H NMR (400 MHz, $CDCl_3$) spectrum of 6,7-Dipentoxycoumarin **(47)**

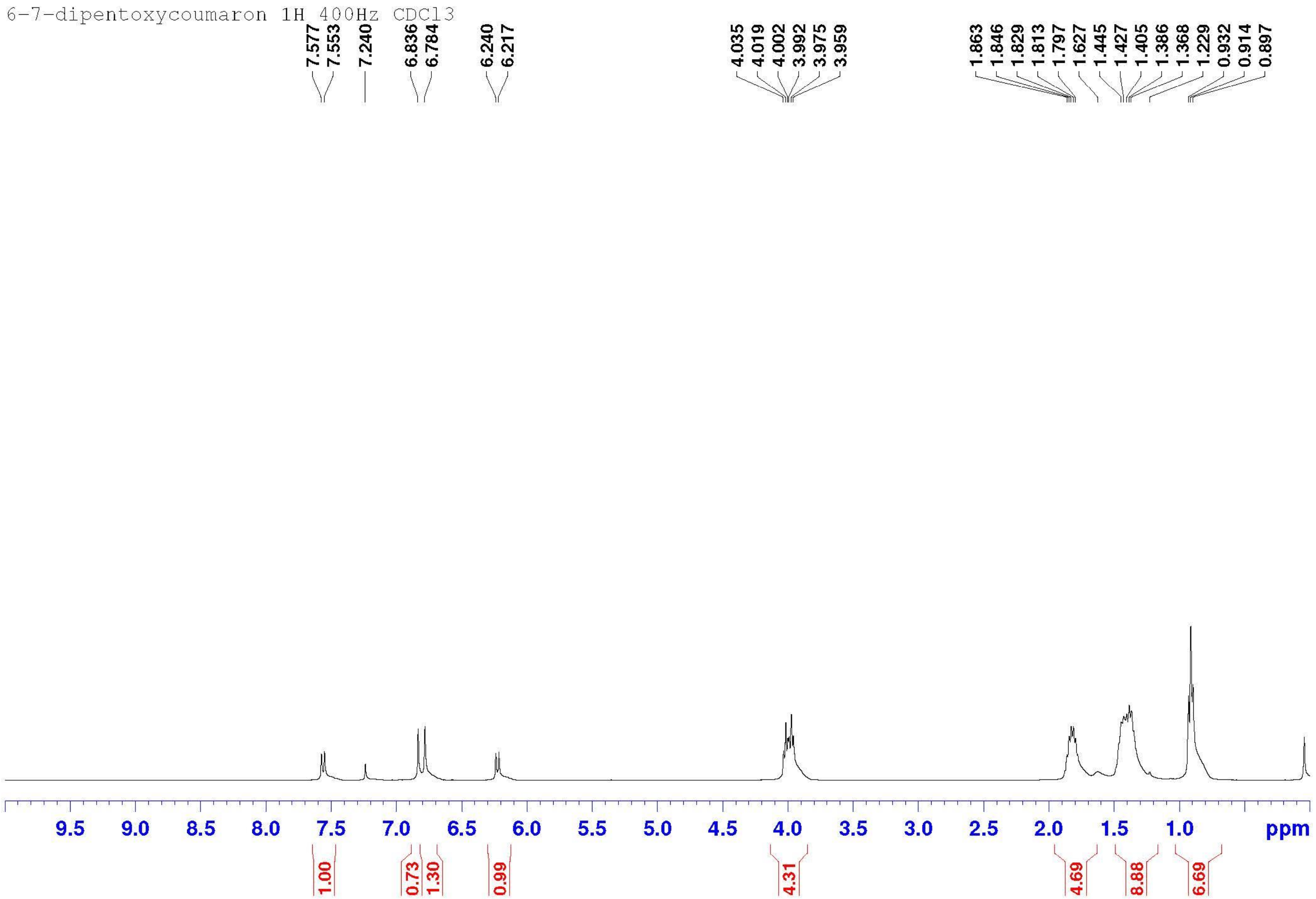

S14 ^{13}C NMR (100 MHz, $CDCl_3$) spectrum of 6,7-Dipentoxycoumarin **(47)**

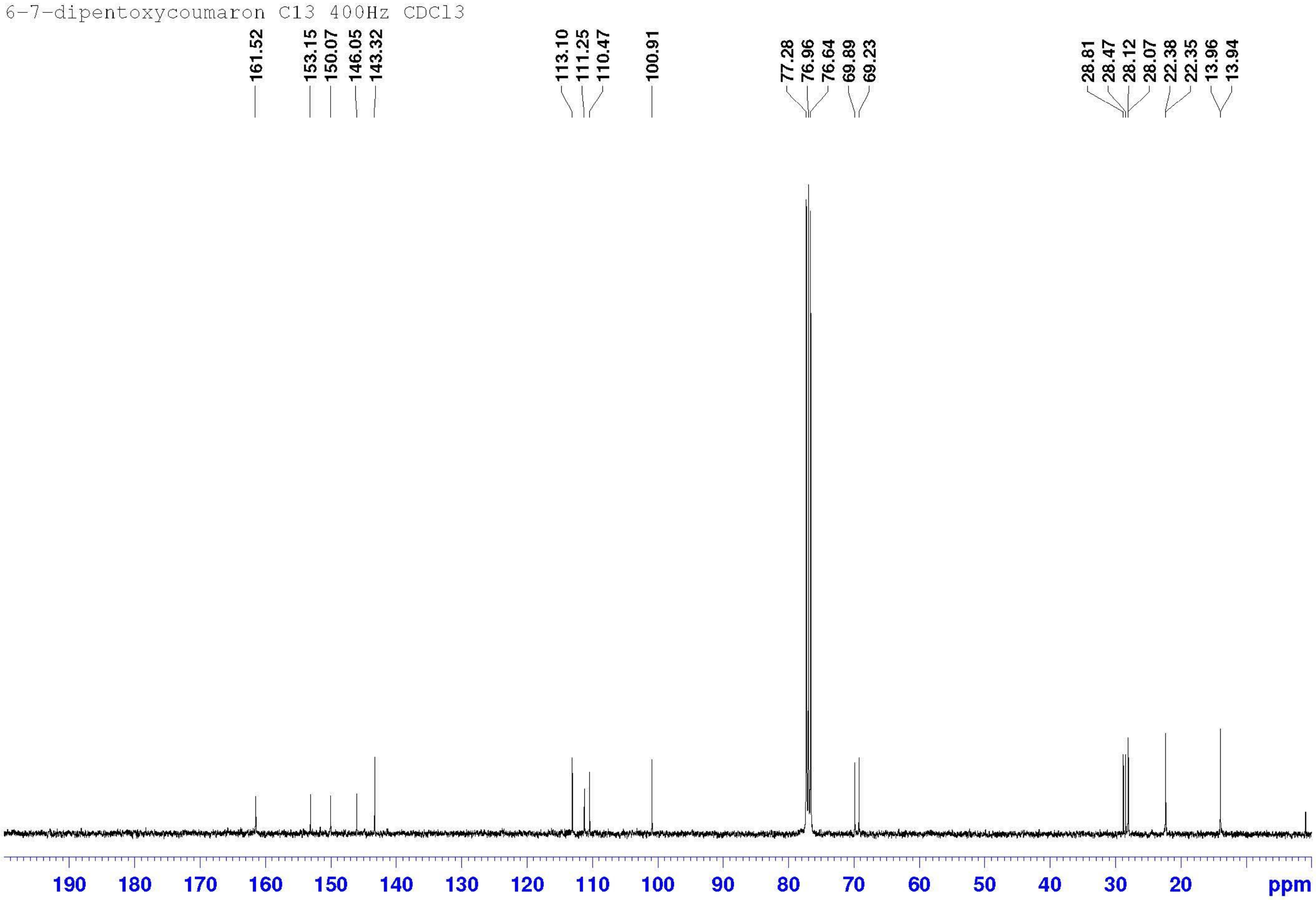

S15 ^{1}H NMR (400 MHz, $CDCl_3$) spectrum of 6,7-Di-isopentoxycoumarin **(48)**

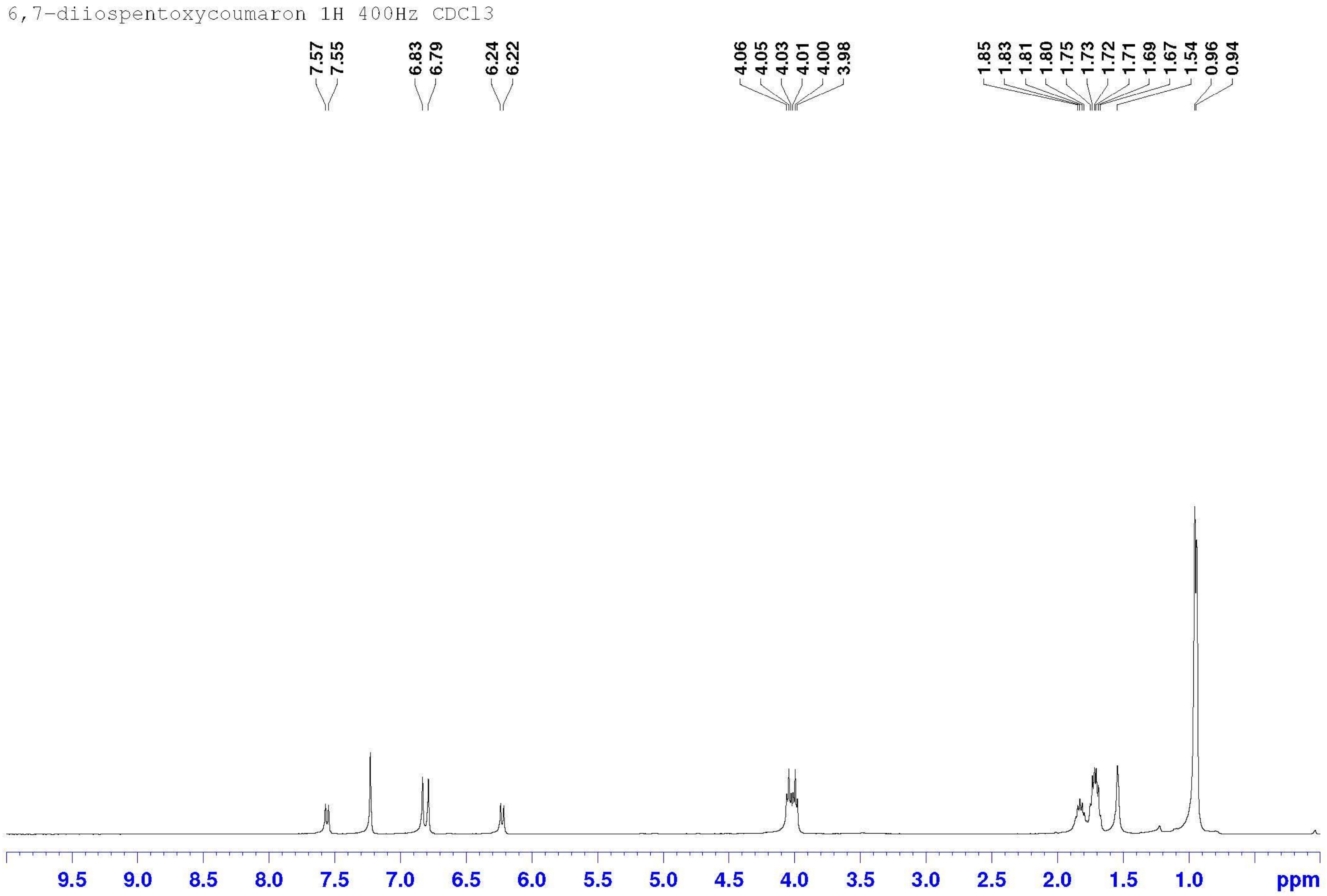

S16 ^{13}C NMR (100 MHz, $CDCl_3$) spectrum of 6,7-Di-isopentoxycoumarin **(48)**

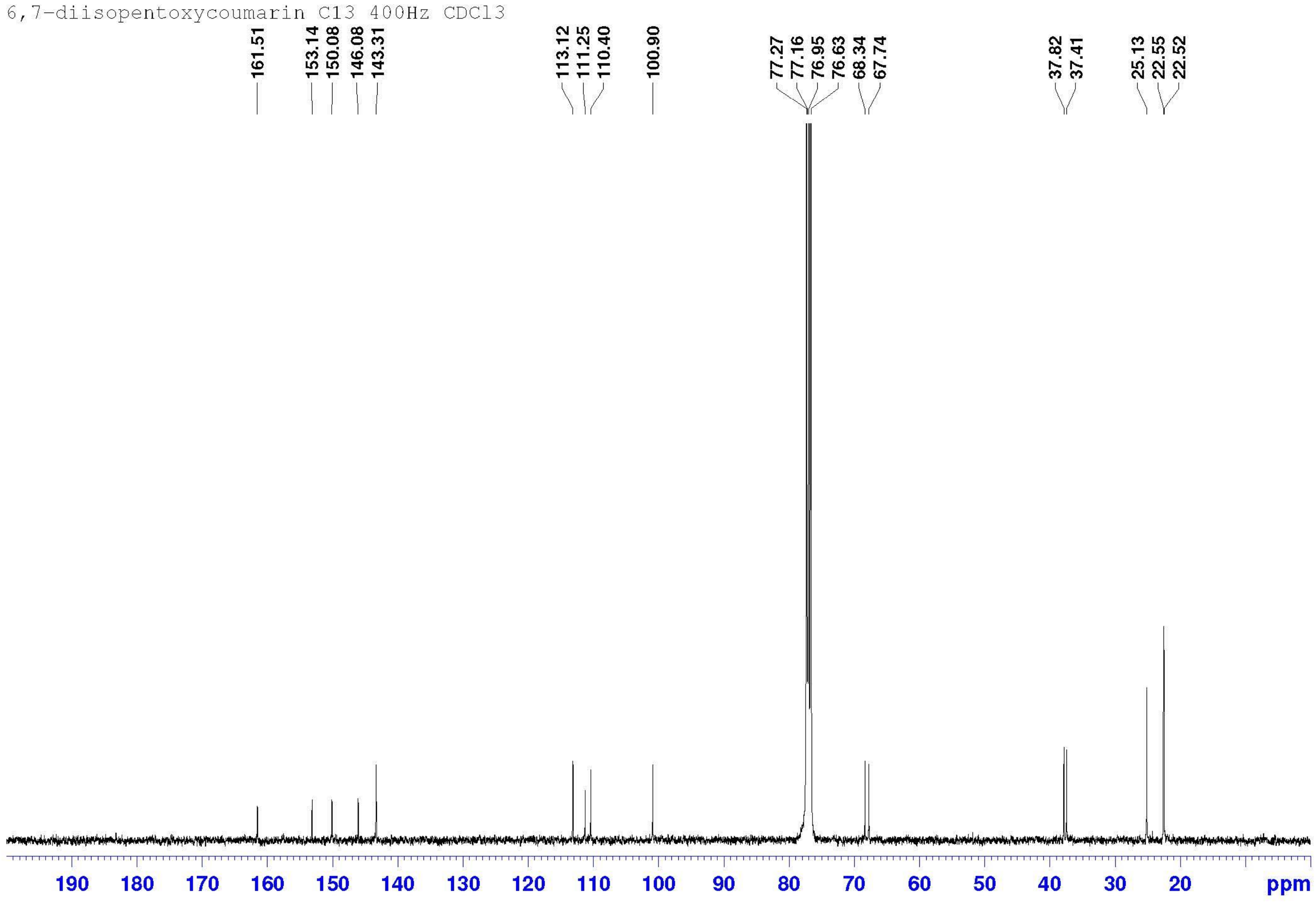

S17 ^{1}H NMR (400 MHz, $CDCl_3$) spectrum of 6,7-Dihexoxycoumarin **(49)**

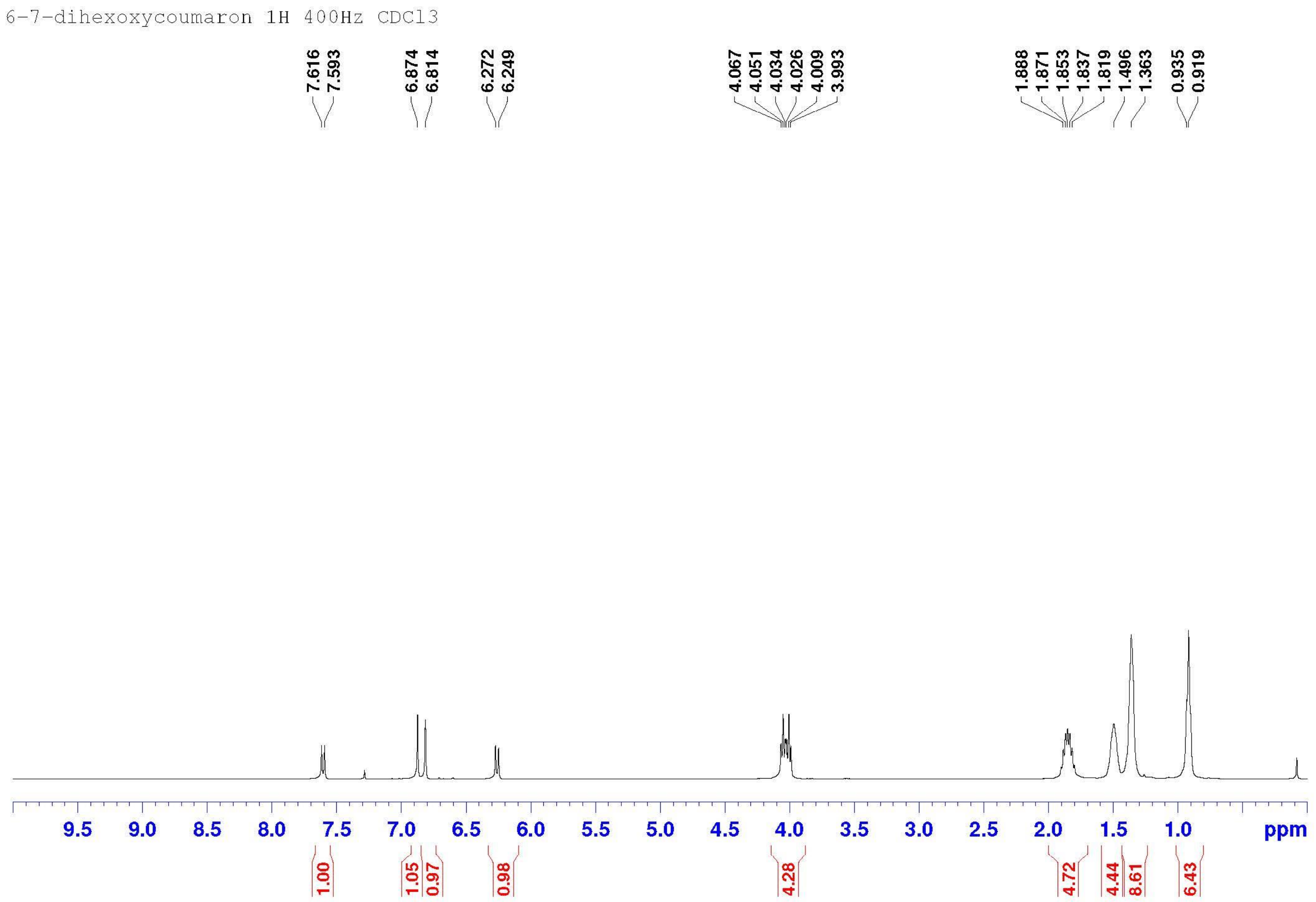

S18 ^{13}C NMR (100 MHz, $CDCl_3$) spectrum of 6,7-Dihexoxycoumarin **(49)**

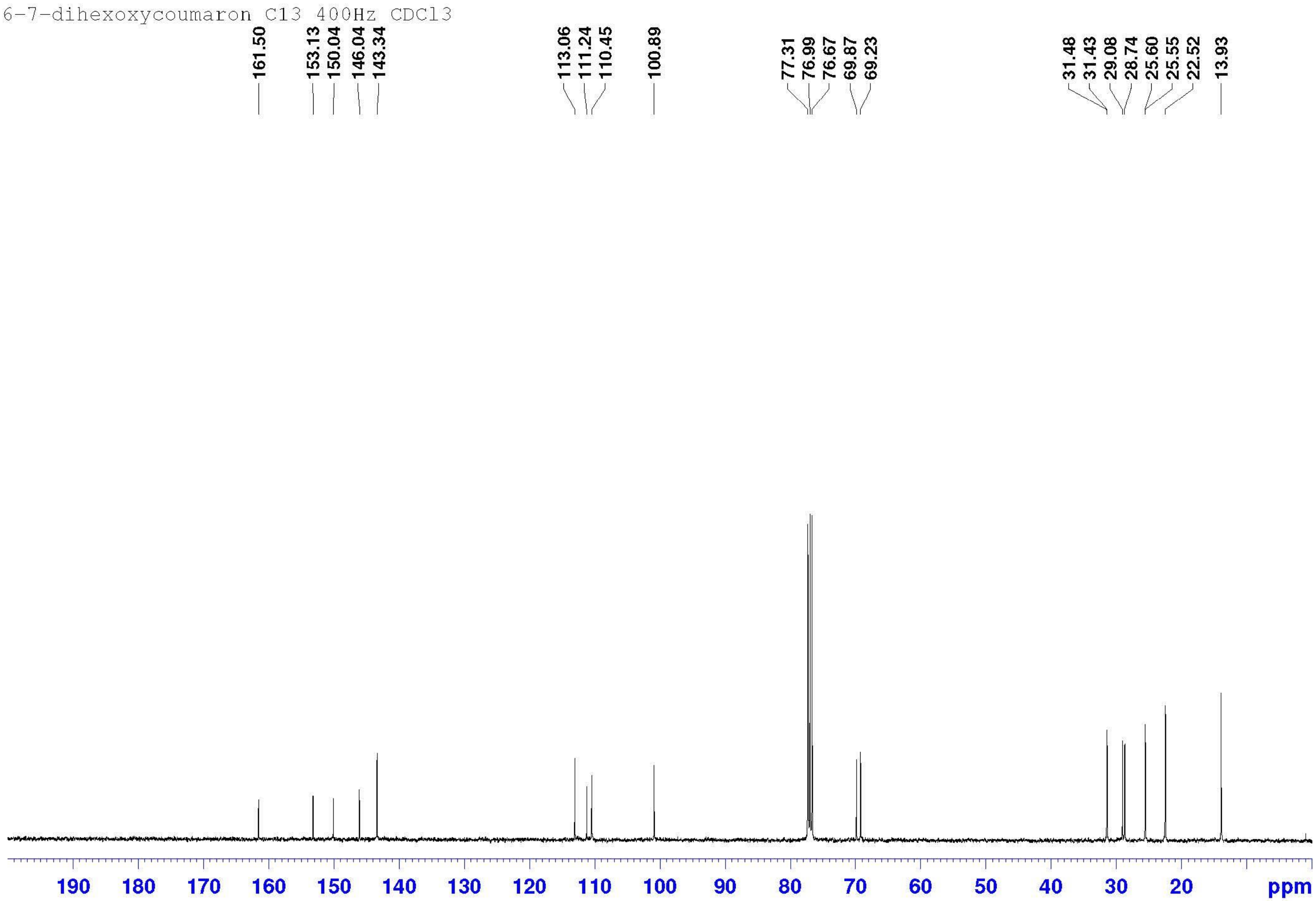

S19 ^{1}H NMR (400 MHz, $CDCl_3$) spectrum of 6,7-Digeranoxycoumarin **(51)**

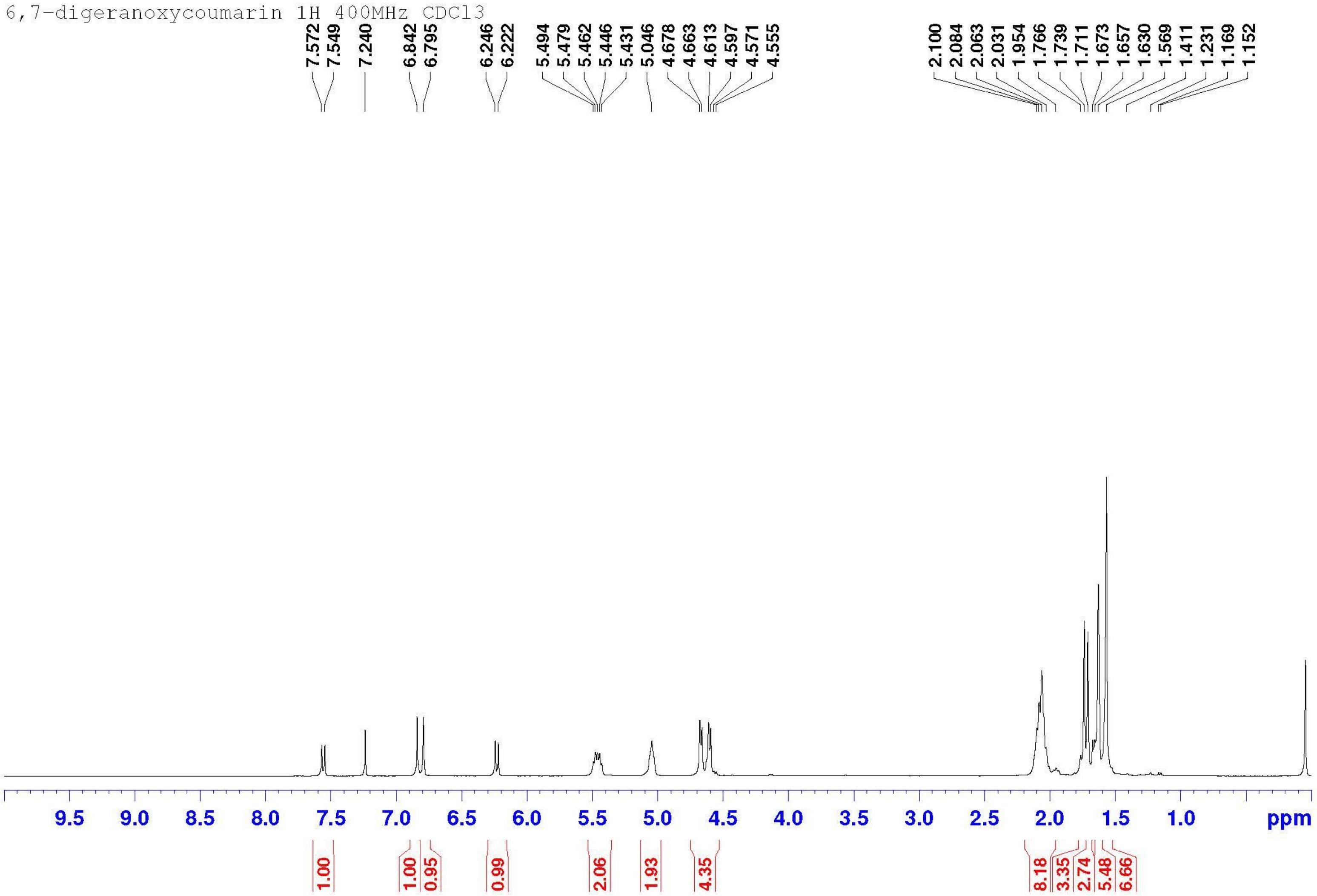

S20 ^{13}C NMR (100 MHz, $CDCl_3$) spectrum of 6,7-Digeranoxycoumarin **(51)**

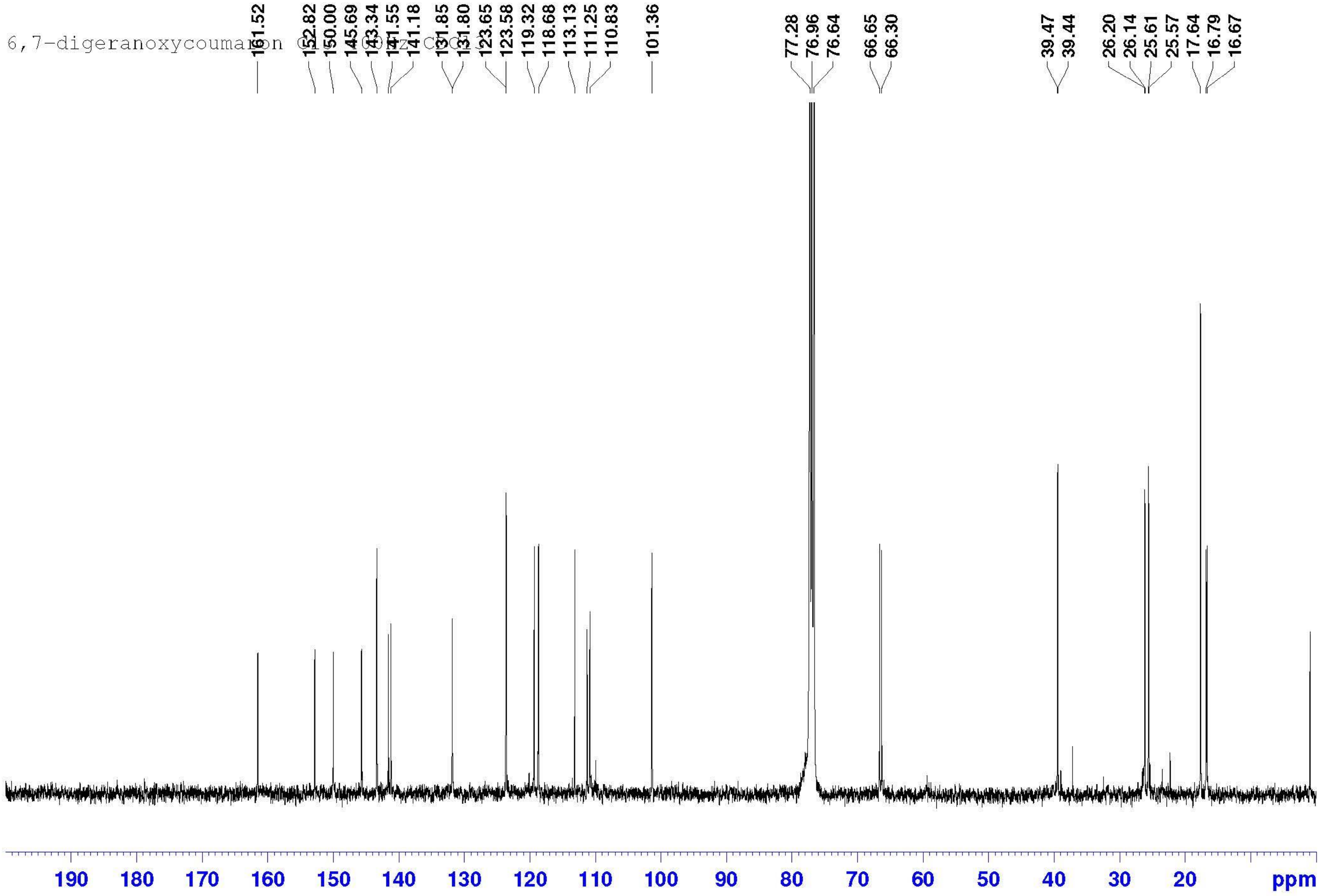

S21 Effects of scoparone, **50 (PLH)**, and **6** on phosphoenopyruvate carboxykinse (PEPCK) mRNA expression induced by dexamethasone and 8-bromo-cAMP.

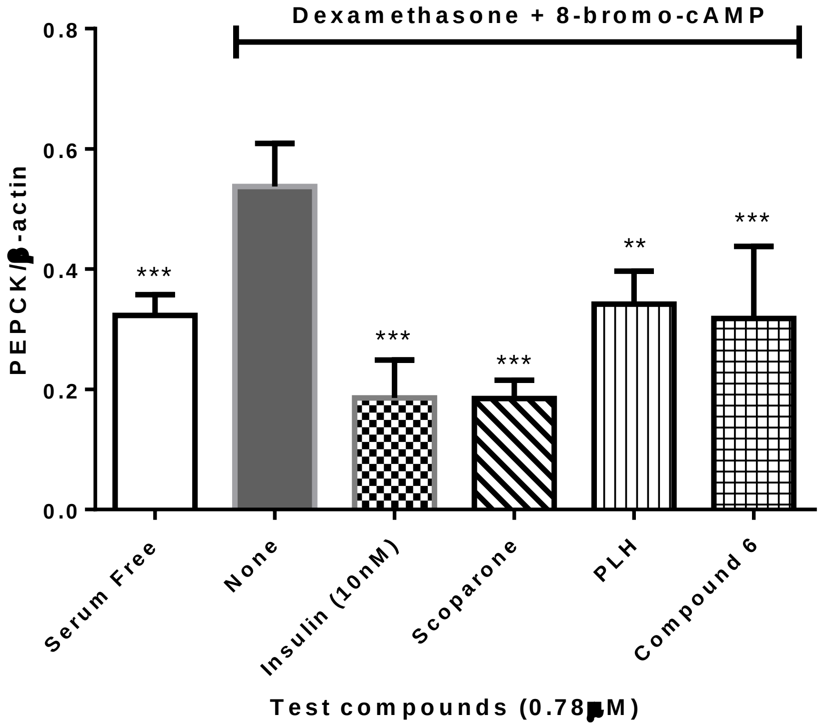

A quantified result of dexamethasone (500 nM) and 8-bromo-cAMP (100μM) induced PEPCK mRNA expression in the presence or absence of scoparone, **50** (PLH) and **6** at 0.78 μM was shown. Insulin (10 nM) was served as an experimental control. Quantified data was presented as mean ± S.E.M. (n = 4). **P < 0.01 and ***P < 0.001 when compared with induction control (None).

Results

PEPCK mRNA could be induced by dexamethasone and 8-bromo-cAMP after 3hr. The presence of insulin could over dominantly suppress such induction (p<0.01). Scoparone, at 0.78 μM, can suppress PEPCK mRNA induction at the level similar to insulin. In contrast, both **50** (PLH) and **6** could also suppress PEPCK expression at the concentration of 0.78 μM but appeared to be less potent.

Methods

Cell culture: The rat H4IIE cells were cultured in Dulbecco's Modified Eagle'sMedium (DMEM) containing 1 g/L glucose, 5% (v/v) fetal calf serum (FBS) and 1% (v/v) antibiotics (100 U/mL penicillin and 0.1 g/L streptomycin). Cultured cells were maintained at 37 ◦C in an atmosphere of 5% CO2 and 95% air.

The measurement of PEPCK gene expression: H4IIE cells were seeded into 6 cm dish and allowed for the attachment. After serum starvation overnight, cells were treated with various conditions described in the figure legend. After treatment, total RNA of each treatment was extracted using TRI-reagent according to the manufacturer's instructions. The pelleted RNA was dissolved in distilled water, quantified with NanoDrop™ 1000 Spectrophotometer (Thermo Fisher Scientific, USA) and stored at −70 ◦C. Total RNA (1μg) was reverse transcribed to generate templates. 50 ng of cDNA was employed for further PCR. The sequences of primers for β-actin: 5'-CGTAAAGACCTCTATGCCAA- 3' and 5'-AGCCATGCCAAATGTGTCAT-3' and

for PEPCK: 5'- AAGGCCGCACCATGTATGTC-3' and 5'-AGCAGTGAGTTCCCACCGTAT- 3'. In addition, annealing temperature for amplification of β-actin (57 ◦C) or PEPCK (57 ◦C) was employed to generate 349 and 319 b.p., respectively. Once the reaction was completed, PCR products were separated by gel electrophoresis, visualized and photographed with a digital camera, and quantified with Genetools 3.06 (Syngene, Frederick, USA).

S22 Treatment of scoparone on db/db mice for two weeks improves glucose tolerance and insulin sensitivity.

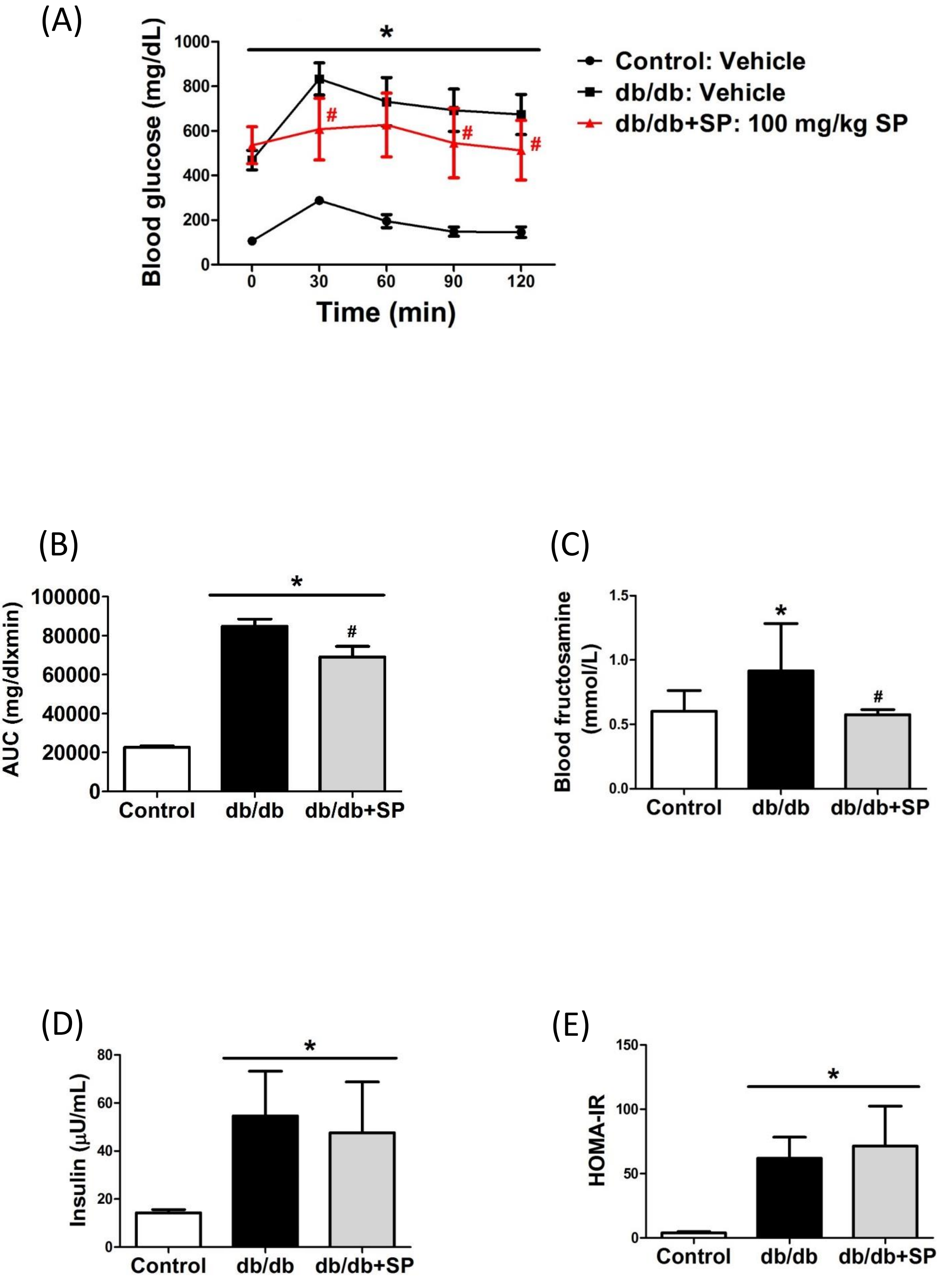

Treatment of scoparone on db/db mice for two weeks improves glucose tolerance. (A) db/db mice were treated with scoparone or vehicle (P.O.) for 2 weeks. After 12 h of fasting, glucose tolerance tests were performed and blood glucose level were assessed. (n= 6) (B) Area under curve of blood glucose level were calculated. Fructosamine (C) and insulin (D) levels were measured. Homeostasis Model Assessment

(HOMA) was calculated as HOMA-IR index= insulin（μU/mL）× glucose（mmol/L）/22.5.

Results

Db/db mice are diabetes animal model characterized by genetically obese leptin receptor-deficient, hyperglycemia and hyperinsulinemia. These mice were treated with scoparone (100 mg/kg, P.O.) or vehicle (CMC) for two weeks. Scoparone-treated group showed a significant improvement in oral glucose tolerance test (OGTT), which was not observed in vehicle group (A). Further analysis showed the level of fructosamine, a glycation end product, also decreased after treatment, indicating blood glucose level became lower after treatment (B). However, insulin level (C) or HOMA-IR (D) didn't not change, indicating that glucose-lowering effect was not due to insulinotropic effect.

Methods

Animals

Six-week old male C57/BL6 mice (n=6) were acquired from the Animal Center of the College of Medicine, National Taiwan University, Taipei, Taiwan and 6-week-old male *db/db* mice (BKS.Cg-Dock7m +/+ Leprdb/JNarl) (n=12) were acquired from the National Laboratory Animal Center, Tainan, Taiwan. Experimental animals were allowed to acclimate to the controlled photoperiod (a cycle of 12-hr light/ 12-hr dark), humidity (40-60% relative humidity) and temperature (22 ± 2 °C) with *ad libitum* supply of standard chow diets and drinking water for one week prior to the experimental treatment in accordance with the Guide for the Care and Use of Laboratory Animals (Institute of Laboratory Animal Resources Commission on Life Sciences, 2011). Acclimated animals were randomly separated into three cohorts: control group (C57/BL6 mice treated with vehicle for two weeks, n=6), db/db group (*db/db* mice treated with vehicle for two weeks, n=6), and db/db+SP group (*db/db* mice treated with 100 mg/kg scoparone for two weeks, n=6).

Ref:

Institute of Laboratory Animal Resources Commission on Life Sciences, National Research Council. In Guide for the Care and Use of Laboratory Animals, 8th ed.; National Academy Press: Washington, DC, 2011.

Oral glucose tolerance test (OGTT) The control mice or diabetic mice with or without drugs treatment for 2 weeks received an oral glucose challenge (2 g/kg). Mice were under slight anesthesia by an intraperitoneal injection of pentobarbital (50 mg/kg) and blood samples were collected following: 0, 15, 30, 60, 90, and 120 min after delivery of the glucose load. Blood glucose levels were determined using SURESTEP blood glucose meter (Lifescan). Glucose tolerance was determined and performed as the curve (AUC) and delta AUC (ΔAUC) using the GraphPad Prism 5 software.

Determination of blood insulin and fructosamine. To determine the amount of insulin and fructosamine after fasting overnight, blood samples of sacrificed animals were collected and analyzed by insulin and fructosamine immunoassay kits according to the respective instructions of the manufacturer (Mercodia AB Inc., Uppsala, Sweden; Hospitex diagnostics Lp, League City, TX, USA). The homeostasis model assessment of insulin resistance (HOMA-IR) index is calculated with the formula of the values of fasting glucose and insulin divided by 22.5 as previously described (Wallace et al., 2004).

Ref:

Wallace TM, Levy JC, Matthews DR. Use and abuse of HOMA modeling. Diabetes Care. 2004;27(6):1487-

95.

Statistical analysis The values in the graph are given as mean±S.E.M. The significance of difference was evaluated by the paired Student's t-test. When more than one group was compared with one control, significance was evaluated according to one way analysis of variance (ANOVA). Probability values of <0.05 were considered to be significant.

S23 Preliminary pharmacokinetic studies of scoparone and 6,7-diprenoxycoumarin

Table S1 Blood concentration of scoparone.

	mouse	Dose given (mg)	Body weight (g)	Time (min)	Cal. Conc. (μg/ml)
Study 1	1	0.2	20.00	10	0.41
	2	0.2	19.12	60	0.26
	3	0.2	19.90	315	0.04
Study 2	1	0.5	20.50	10	10.66
	2	1.1	20.02	60	9.22
	3	0.5	20.75	320	0.04

Table S2 Blood concentration of 6,7-diprenoxycoumarin.

	mouse	Dose given (mg)	Body weight (g)	Time (min)	Cal. Conc. (μg/ml)
Study 1	1	0.36	23.4	10	0.56
	2	0.36	22.3	60	0.08
	3	0.36	23.9	110	0.07
	4	0.36	23.16	360	0.04
Study 2	1	0.5	20.92	11	0.31
	2	0.5	21.94	60	0.12
	3	0.5	19.94	318	0.027

Results

Adopt previous study on fraxetin (unpublished result), a coumarin compound, RP-HPLC-fluorescence method (excitation 380 nm and emission 480 nm) was applied on analysis of the concentration of scoparone and 6,7-diprenoxycoumarin. These chromatograms revealed that the test compounds can be clearly analyzed without interference with endogenous plasma constituents. Calibration curve of scoparone showed retention time of approximately 7.56 min in mouse plasma, while calibration curve of 6,7-diprenoxycoumarin showed retention time of approximately 7.51 in mouse plasma. The calibration curves showed good linearity from 0.01 to 10.00 μg/ml for scoparone and 0.025 to 6.25 μg/ml for 6,7-diprenoxycoumarin. The relative error and coefficient of variance are all below 15 %. The LOQ were 0.01 and 0.005 μg/ml for scoparone and 6,7-diprenoxycoumarin. Linearity over the investigated concentration was obtained.

After method validation, C57BL/6 mice were given scoparone (i.p.) or 6,7-diprenoxycoumarin and plasma concentration-time were presented in Table S1 and S2. In pharmacological assay, the desired scoparone concentration is ~ 2 μg/ml (10 μM), which can be achieved when dose given was 0.5 mg/mice in Table S1. However, the desired 6,7-diprenoxycoumarin concentration (~ 3 μg/ml, 10 μM) cannot be obtained even if the amount of drug increased to 0.5 mg/mice. Thus, the CAR activating effect may not be seen in vivo, which can account for no blood sugar lowering effects of 6,7-diprenoxycoumarin in vivo.

Methods

Chemicals and Reagents

Acetonitrile HPLC-grade (LiChrosolv®) were purchased from Sigma-Aldrich Chemie GmbH. Deionized water was purified on a Milli-Q system. Scoparone and 6,7-diprenoxycoumarin were synthesized from our

lab and purity was > 95 % from HPLC-UV method.

Instrumentation and Chromatographic Condition

Hitachi L-7100 liquid chromatography system (Hitachi, Japan) comprised a Hitachi D-7000 interface, a Hitachi L-7200 auto sampler, a Hitachi L-7400 UV detector and a Hitachi L-2480 FL detector. The HPLC colum consisted of a Thermo Hypurity® RP-18 column (200 × 4.6 mm, 5 μm) connected to a Hichrom ODS guard column. The isocratic elution was applied for scoparone (ACN-H_2O = 3:7) and 6,7-diprenoxycoumarin (ACN-H_2O = 7:3). The flow rate was set at 1.0 ml/min. The detection fluorescence was set at excitation 380 nm and emission 480 nm. A volume of 20 μL of sample was subjected to HPLC for analysis.

Animals and Sample Preparation

Eight-week old male or female C57/BL6 mice were acquired from BioLASCO Taiwan Co., Ltd. Experimental animals were allowed to acclimate to the controlled photoperiod (a cycle of 12-hr light/ 12-hr dark), humidity (40-60% relative humidity) and temperature (22 ± 2 °C) with *ad libitum* supply of standard chow diets and drinking water for one week prior to the experimental treatment in accordance with the Guide for the Care and Use of Laboratory Animals (Institute of Laboratory Animal Resources Commission on Life Sciences, 2011). Acclimated animals were randomly separated into two groups: scoparone-treated or 6,7-diprenoxycoumarin-treated. Mice were administered indicated dose i.p. All mice were anesthetized with Zoetil (10 mg/ml, 0.05 ml/10 gmbw, i.p.) before blood sampling. The blood samples (approximately 300 μL) were collected under heparin treatment from heart at indicated time points shown in the table S1 and S2. After centrifuging at 10,000 rpm for 10 min, the plasma samples were obtained. The plasma (50 μL) was mixed with 150 μL ACN, vortexed, and centrifuged for 130,000 rpm, 10 mins and at 4 °C. The supernatant was obtained and 20 μL of sample was subjected to HPLC analysis.

Ref:

Institute of Laboratory Animal Resources Commission on Life Sciences, National Research Council. In Guide for the Care and Use of Laboratory Animals, 8th ed.; National Academy Press: Washington, DC, 2011.
